# Intermediate and high-risk non-muscle-invasive bladder cancer: an overview of epidemiology, burden, and unmet needs

**DOI:** 10.3389/fonc.2023.1170124

**Published:** 2023-06-02

**Authors:** Kristin Grabe-Heyne, Christof Henne, Paramananthan Mariappan, Götz Geiges, Johannes Pöhlmann, Richard F. Pollock

**Affiliations:** ^1^ Medac GmbH, Wedel, Germany; ^2^ Edinburgh Bladder Cancer Surgery (EBCS), Department of Urology, Western General Hospital, Edinburgh, United Kingdom; ^3^ Praxis Dr. Geiges, Berlin, Germany; ^4^ Covalence Research Ltd., Harpenden, United Kingdom

**Keywords:** non-muscle-invasive bladder cancer (NMIBC), Bacillus Calmette-Guérin (BCG), treatment adherence, guideline compliance, radical cystectomy, unmet need, data gap

## Abstract

Bladder cancer ranks among the most common cancers globally. At diagnosis, 75% of patients have non-muscle-invasive bladder cancer (NMIBC). Patients with low-risk NMIBC have a good prognosis, but recurrence and progression rates remain high in intermediate- and high-risk NMIBC, despite the decades-long availability of effective treatments for NMIBC such as intravesical Bacillus Calmette-Guérin (BCG). The present review provides an overview of NMIBC, including its burden and treatment options, and then reviews aspects that counteract the successful treatment of NMIBC, referred to as unmet treatment needs. The scale and reasons for each unmet need are described based on a comprehensive review of the literature, including insufficient adherence to treatment guidelines by physicians because of insufficient knowledge, training, or access to certain therapy options. Low rates of lifestyle changes and treatment completion by patients, due to BCG shortages or toxicities and adverse events as well as their impact on social activities, represent additional areas of potential improvement. Highly heterogeneous evidence for the effectiveness and safety of some treatments limits the comparability of results across studies. As a result, efforts are underway to standardize treatment schedules for BCG, but intravesical chemotherapy schedules remain unstandardized. In addition, risk-scoring models often perform unsatisfactorily due to significant differences between derivation and real-world cohorts. Reporting in clinical trials suffers from a lack of consistent outcomes reporting in bladder cancer clinical trials, paired with an under-representation of racial and ethnic minorities in many trials.

## 1 Introduction

Bladder cancer imposes a substantial burden on patients, healthcare systems, and societies across the globe (1, 2). At diagnosis, approximately 75% of bladder cancer cases are non-muscle-invasive bladder cancer (NMIBC), which is a clinically heterogeneous disease characterized by high recurrence and, in high-risk disease, high progression rates (3–5). Much of the burden of NMIBC persists despite the decade-long availability of effective treatments such as Bacillus Calmette-Guérin (BCG) immunotherapy (6, 7). The NMIBC burden will likely increase due to population aging (2, 8, 9). It is therefore important to investigate the reasons for the current scale of the burden to identify the levers with which clinicians and healthcare decision-makers can improve NMIBC care.

In the present study, we provide a review of bladder cancer and NMIBC, including disease classification, epidemiology, humanistic and economic burden, and treatment options. We focus on patients with intermediate- and high-risk NMIBC (defined below). These patient groups have unmet needs regarding physician adherence to treatment guidelines, as well as lifestyle changes and treatment completion rates. Existing evidence is also often of limited value due to unstandardized treatment schedules and risk stratification systems that perform suboptimally in clinical practice. The paper reviews these unmet treatment and evidence needs and highlights where improvements in treatment use, patient care, and evidence generation could be helpful to achieve better outcomes for patients with intermediate- and high-risk NMIBC.

## 2 Results

### 2.1 NMIBC: staging, grading, risk, and outcomes

#### 2.1.1 Staging

Bladder cancer is staged using the American Joint Committee on Cancer and Union for International Cancer Control staging classification, based on tumor size (T), lymph node (N) involvement, and presence of distant metastasis (M) (10). The umbrella term NMIBC includes tumors at stages Ta (confined to the mucosa), T1 (invading the lamina propria), and Tis (carcinoma *in situ*; flat, high-grade tumor confined to the mucosa) (11). This contrasts with bladder cancer which has already invaded the muscle (MIBC) and with metastatic disease.

#### 2.1.2 Grading

Histological grading uses grading systems developed by the World Health Organization (WHO). Until 2004, the WHO 1973 grading system was used, which differentiates papillary urothelial lesions into three grades. In 2004, the classification was revised (with another revision in 2016) to address the problems of the 1973 system. The twice-revised 2004/2016 WHO classification distinguishes papillary urothelial neoplasm of low malignant potential, low-grade, and high-grade tumors (12, 13). The European Association of Urology (EAU) currently recommends using both the three-tier 1973 (G1, G2, G3) and the two-tier 2004/2016 system (low-grade [LG], high-grade [HG]), resulting in a four-tier grading system (LG/G1, LG/G2, HG/G2, and HG/G3) (11, 12). The four-tier system outperforms its constituent systems as it splits the relatively large group of patients with G2 disease into patients with LG and HG disease who have different prognoses (14). Grading is prognostic for disease progression, but not recurrence (11).

#### 2.1.3 Risk stratification

A patient’s recurrence and progression risk should inform their treatment. Patient risk is estimated using prognostic models, which allow stratifying patients into risk groups, based on clinical and pathological characteristics (11, 15, 16). Such models and accompanying stratification tables listing the clinical features of each risk group are developed by guideline-issuing organizations such as the EAU and the American Urological Association (AUA) and independent research groups. There are many scoring models and risk stratification tables, which are similar in structure and output but differ in data sources (15–21). In particular, derivation cohorts differ in the type of surgical and intravesical treatment received, which may bias risk estimates and reduce the discriminative ability of models in populations with different treatment experiences (22–28). Clinicians are faced with the challenge of selecting from the many available tools, all of which have specific shortcomings and may not fit their patient population.

An early scoring model was developed on behalf of the European Organisation for Research and Treatment of Cancer (EORTC) (18, 29). The model was developed from individual-level data of 2,596 patients with Ta/T1 bladder cancer enrolled in seven EORTC trials. Recurrence and progression were predicted using tumor number, size, stage, grade, and status (primary or recurrent, including time to recurrence), and presence. In response to perceived shortcomings of the EORTC model, in particular the absence of patients treated with BCG in the derivation cohort, the Spanish Club Urológico Español de Tratamiento Oncológico (CUETO) developed a new risk stratification model (15). The model was designed to account for to account for recurrence and progression following use of BCG and derived from data of 1,062 patients receiving BCG after a transurethral resection of the bladder (TURBT) in four CUETO-sponsored trials. The predictors used were similar to those in the EORTC model and included age, gender (for recurrence prediction), as well as tumor status (primary or recurrence), number, stage, and grade, and presence of carcinoma *in situ* (Cis). Relative to the EORTC model, lower recurrence and, for patients with high-risk tumors, lower progression risks were obtained in the CUETO model (15, 30).

Subsequently, an EORTC nomogram was developed to predict recurrence and progression in patients treated with standard 1 to 3 years BCG maintenance therapy (versus the much shorter maintenance therapy used in the CUETO model derivation cohort) (31). The derivation cohort comprised 1,812 patients with Ta/T1 NMIBC (not Cis) from two EORTC trials who had received 1 to 3 years of maintenance therapy with BCG. The nomogram included tumor grade and number and prior recurrence. It showed considerable heterogeneity in time to first recurrence and time to progression among patients receiving BCG maintenance. One of the latest risk models was developed on behalf of the EAU, for predicting time to progression, based on data from patients with Ta/T1 NMIBC treated with TURBT and subsequent intravesical chemotherapy (16). The derivation cohort included 5,145 patients. The model included age, tumor number, stage, and maximum diameter, tumor grade based on either the WHO 1973 or the WHO 2004/2016 grading system, and presence of Cis as predictors. The model introduced the notion of a very high-risk group that should be investigated for prompt treatment.

#### 2.1.4 Clinical outcomes in NMIBC

Clinical outcomes differ across risk groups and countries, as well as by treatment and healthcare system.

A recent systematic review of real-world evidence studies of patients with high-risk NMIBC found 5-year recurrence-free survival (RFS) to vary between 17–89%, while progression-free survival (PFS) varied between 58–89%, cancer-specific survival between 71–96%, and overall survival (OS) between 28–90% (5). In this review, patients who received and completed intravesical therapy with Bacillus Calmette-Guérin (BCG) were found to have longer survival relative to patients not receiving or completing intravesical therapy. Additional data on patients with intermediate- and high-risk NMIBC treated with adequate BCG therapy (32, 33) confirmed excellent survival outcomes over 5 years, with RFS of 86% and 72%, PFS of 100% and 91%, and OS of 79% and 87% in intermediate-risk and high-risk patients, respectively (3). Similarly, following adequate BCG induction treatment, a 10-year PFS of 78% was reported for a US cohort of patients with high-risk NMIBC (34).

More broadly, median survivorship estimates of 5.3 years (95% confidence interval [CI] 5.2 to 5.4 years) and 4.4 years (95% CI 4.3 to 4.6 years), respectively, were reported for US patients between 1988 and 2006 (35). In this US analysis, survivorship differed by age, sex, and race/ethnicity, with non-white and older patients experiencing shorter survival. In Sweden, 59% of patients with intermediate-risk NMIBC experienced a recurrence over a median follow-up of 63 months (36).

### 2.2 Epidemiology and etiology

#### 2.2.1 Global and regional epidemiology of bladder cancer

Epidemiological data specific to NMIBC are sparse but can be approximated from data for bladder cancer, approximately 75% of which are NMIBC at presentation (37). Western and Southern Europe had the highest age-standardized incidence rates (ASIR) of bladder cancer (and, by extension, NMIBC) in 2020. Central America, West and Middle Africa, and parts of Central and Eastern Europe had the lowest ASIR (38). On a per-country level, the highest ASIR were for European countries, including Greece, the Netherlands, and Italy. Outside Europe, the incidence was highest in Egypt.

Bladder cancer incidence has decreased in many parts of the world. The notable exception, for both sexes, is Europe. Regarding men, five of six countries with statistically significant increases in bladder cancer incidence as per the analysis by Teoh et al. (8) were in Europe (plus Japan). Regarding women, eleven countries saw incidence increases, of which ten were in Europe (plus Japan).

#### 2.2.2 Risk factors

Several factors increase the risk of bladder cancer, of which age is the most important. With a median age at diagnosis of approximately 70 years, bladder cancer is primarily considered a disease of the elderly (2, 39), and its incidence increases with age. Data from California, for example, indicated that age-specific incidence peaked in the 85–95-year age group (40). The link between bladder cancer incidence and age results from longer cumulative environmental exposure to carcinogens and more time available for the development and accumulation of cellular events triggering neoplastic transformations. Higher age might also reduce the ability to empty the bladder completely, so carcinogens excreted in urine remain in longer contact with the bladder. Furthermore, difficult voiding might lead older people to drink less, increasing the concentration of carcinogens in the urine (39).

Sex is another key risk factor for bladder cancer incidence and mortality. Men have a considerably higher risk of developing bladder cancer, which is reflected in men dominating the gender split in most clinical and epidemiological studies (41). This gap persists even if accounting for differential exposure to risk factors and may be attributable to differences in carcinogen metabolism and sex steroid hormones (41, 42). Outcomes are not necessarily worse for men, however, as women present later, with more advanced disease. This may indicate a diagnostic gap, particularly when evaluating hematuria, and treatment that disfavors women (41–43).

A key behavioral risk factor is smoking (44). Population-attributable risks of bladder cancer to cigarette smoking range from 20% in China to 37% in Europe (45). An estimated 37% of global bladder cancer disability-adjusted life-years in 2019 were attributable to smoking (2). Lifetime exposure to second-hand smoke may also elevate the risk of bladder cancer (46). A longer smoking duration and more pack-years are associated with an increased NMIBC recurrence risk, suggesting a dose-dependent link between smoking and recurrence (47).

Diet has also been investigated for its role in bladder cancer. Multiple studies have investigated different aspects of diet, including alcohol consumption, vitamin intake, levels of fluid consumption including coffee and tea, vegetable and fruit consumption, meat consumption and the consumption of a Mediterranean diet (48–50). There is some evidence that a diet rich in red and processed meat is associated with an increased risk of bladder cancer (49, 51, 52) while a Western diet may be associated with higher risks of NMIBC recurrence (50). Mono-unsaturated fatty acids may be associated with reduced NMIBC risk and, in men, there may be a positive association between total cholesterol intake and bladder cancer risk (53). The consumption of sugar-sweetened beverages has also been reported to be associated with overall survival in NMIBC, as has sodium consumption for bladder cancer-specific survival (54). The overall diet quality, however, was not associated with either overall survival or bladder cancer-specific survival. Overall, the evidence base for many dietary risk factors remains limited as many studies are small, possibly biased, or hard to interpret due to conflicting results between reports (44). Cohorts with bladder cancer have also been shown to be less physically active than the age-matched general population, with reduced physical activity levels associated with higher body mass index (BMI) and increased smoking exposure (55).

Efforts into researching the prognostic and potentially therapeutic role of the microbiome in bladder cancer are also increasing. Based mostly on tissue and urine samples, the abundance of different genera between healthy people and those with bladder cancer or NMIBC are investigated (56, 57). The overall evidence base so far remains limited to small studies, which currently suggests a rather limited role of the microbiome for disease prediction.

### 2.3 Burden of NMIBC

#### 2.3.1 Effect of NMIBC on patient quality of life

Good QoL at the time of NMIBC diagnosis has been reported in multiple studies. In qualitative studies, patients expressed shock and concern, but many quickly adapted to their diagnosis (58, 59). Some patients expressed frustration that their symptoms were initially not taken seriously enough, although few patients suffered from disease symptoms. Most patients were confident about treatment success while finding treatment decision-making demanding and facilitated by open, consistent communication with healthcare professionals (58, 59). These qualitative findings matched those from a quantitative study, which showed that, at diagnosis, QoL in patients with NMIBC was comparable to that of the general population (60).

Treating NMIBC may reduce QoL, at least transiently, but results are inconsistent. Reduced QoL, measured using the Short Form (SF)-36, was observed in patients undergoing TURBT followed by intravesical immunotherapy or chemotherapy at 6 months after TURBT for the SF-36 Physical domain (61). At 12 months post-operation, the physical score had improved to above pre-operative levels, as had the mental health score. In a comparison of BCG, mitomycin-C (MMC), and chemohyperthermia (CHT), all patients had reached baseline QoL at the end of the induction treatment (62). Similarly, no significant changes in QoL over time were observed in a randomized study comparing BCG and gemcitabine for either treatment (63).

Experiences among survivors and adaptation to post-treatment circumstances differ across patients and QoL domains, with most domains improving with time since treatment (59, 64). In general, NMIBC survivors have reduced QoL relative to the general population (65–67). Incontinence and sexual dysfunction affect QoL negatively and limit social and everyday activities (59, 66, 67).

The effect of radical cystectomy on QoL in bladder cancer patients has received particular attention, but findings again are inconsistent. Tsai et al. (68) reported that radical cystectomy was associated with reduced QoL up to 5 years after treatment across the physical, psychological, and social domains of the WHO Quality of Life questionnaire. Health state utility values elicited from the general population, for example by Cooper et al. (69) for the UK, also showed a substantial reduction in QoL following radical cystectomy for BCG-unresponsive NMIBC. In contrast, Clements et al. (70) reported that, within 24 months of radical cystectomy, all QoL domains except sexual function had returned to or surpassed baseline QoL. In an earlier meta-analysis, Yang et al. (71) concluded that QoL may improve after radical cystectomy, but that urinary and sexual dysfunction were persistently worse than in the general population.

Quality of life is reduced by recurrence and progression, but the effect of recurrence requires further investigation. From the United Kingdom (UK) BOXIT trial, statistically significant decrements in QoL were estimated for grade 3 recurrence (but not lower-grade recurrences) and progression to MIBC in high-risk patients (72). Including intermediate-risk patients in the analysis resulted in estimates for recurrence that were no longer statistically significant. A multivariable analysis by Smith et al. (73) also showed no difference in QoL between patients with NMIBC with and without recurrent disease. As in the BOXIT data, progression to MIBC was associated with reductions in the EORTC summary score versus non-recurrent NMIBC, although this relationship was not consistent across all domains.

#### 2.3.2 Economic burden of NMIBC

Assessment of the economic burden of bladder cancer dates back at least to a 2003 health economic review (74). This review of forty-four studies concluded that bladder cancer was associated with the highest per-patient cost of all cancers from diagnosis to death, with, at the time, costs of USD 96,000 to USD 187,000 (2001 values). These costs reflected the long-term survival and associated need for lifelong routine monitoring in patients with bladder cancer. Overall, bladder cancer was the fifth most expensive cancer in the US in terms of total medical expenditure, with direct annual costs of USD 3.7 billion in 2001 values (74). These findings were confirmed in a 2014 review that reviewed costs over the bladder cancer diagnosis, treatment, and surveillance trajectory (75). A sizable portion of the costs associated with bladder cancer management were due to cystoscopic, radiologic, and interventional procedures used to guide treatment decisions, including annual CT scans and routine use of urine cytology. While intravesical therapy was relatively inexpensive, particularly given its ability to prevent costly recurrence, a lack of consensus regarding maintenance therapy at the time meant that BCG maintenance could be improved further to minimize treatment-related toxicity and overall cost-effectiveness. In contrast, radical cystectomy was associated with significant costs, including those of perioperative complications. Surveillance contributed substantially to costs, e.g., cystoscopy performed every 3 months even in patients with low-grade disease (75).

High costs associated with NMIBC specifically were confirmed in a 2020 review (76). Recurrence and progression as well as associated complications of NMIBC were major cost drivers. Cumulative care costs increase nearly threefold from low-risk to intermediate-risk NMIBC, and increase by a factor of 2.5 again from intermediate-risk to high-risk disease in the US (77). As for bladder cancer generally, surveillance was identified as an important contributor to costs, particularly in patients with high-risk NMIBC. Recent studies, however, have suggested that adaptations to existing surveillance schedules would be possible to reduce costs. In Japan, a surveillance schedule distinguishing high-risk from very high-risk patients and employing period-specific recurrence detection rates to inform the necessity of routine surveillance was shown to reduce 10-year surveillance costs by 40% relative to the standard EAU schedule (78).

Regarding the country-specific economic burden of NMIBC, in the US, 2019 per-patient costs for high-risk NMIBC treated in Department of Veterans Affairs centers were estimated at USD 29,459, USD 55,267, and USD 117,361 at 1, 2, and 5 years, respectively (34). Costs in patients with progression were statistically significantly higher than in patients without progression. Outpatient costs were by far the most important cost category.

For the UK, 3-year average costs per patient with NMIBC, for 2017 from the National Health Service perspective, were estimated at GBP 8,735, with cost increments of GBP 1,217 to GBP 3,957 in case of recurrence and GBP 5,407 in case of progression (72). The 3-year total costs of NMIBC in the UK were estimated at GBP 66 million for the cohort diagnosed in 2015.

For Italy, 2016 cost data were available from a cost-of-illness study, in which 62% of patients had NMIBC (79). The median cost per case with NMIBC was EUR 4,584. The highest costs were due to surgical procedures, radiotherapy, and non-intravesical chemotherapy, while intravesical chemotherapy and BCG were the least significant cost items. Total bladder cancer costs in 2016 for Italy were estimated at EUR 1.2 billion, of which 56% were direct costs.

The societal costs of NMIBC survivors over 10 years in Germany were reported as EUR 2,214 for low-risk, EUR 4,758 for intermediate-risk, and EUR 11,325 for high-risk NMIBC in 2020 (80). Patients incurred EUR 606 (low-risk patients), EUR 1,284 (intermediate-risk patients), and EUR 2,931 (high-risk patients) of costs in 2020, with the rest covered by healthcare providers and healthcare insurance. Survivorship costs, highest in the first two years after the first treatment, had increased, with a 65% rise in overall expenditure between 2000 and 2020.

Mortality from bladder cancer also contributes to the economic burden. Productivity losses from premature bladder cancer deaths in Europe were estimated, per premature death, as EUR 117,436 of paid production lost in the employed and as EUR 44,093 and EUR 74,096 of unpaid production lost in the employed and unemployed, respectively (81).

### 2.4 Treatment options for intermediate- and high-risk NMIBC

An ever-increasing number of guidelines for NMIBC treatment have been issued by national and international societies (**Supplementary Tables S1**, **S2**). The quality of these guidelines varies, particularly regarding stakeholder involvement, the rigor of development, and applicability (82).

#### 2.4.1 TURBT

Following patient assessment and work-up, the first step in managing NMIBC is TURBT (11, 17, 83). During TURBT, all visible tumors should be removed (therapeutic goal) and disease staging performed (diagnostic goal). Fractioned or *en-bloc* techniques can be used, based on tumor size and location, and surgeon experience. The relative merits of each technique remain to be established, but *en-bloc* resection may be associated with reduced hospitalization and catheterization duration, without differences in complication rates (83, 84). A surrogate criterion for resection quality and required part of TURBT in all but Ta G1/LG tumors is the presence of detrusor muscle in the specimen. Its absence is associated with significantly higher risks of residual disease, early recurrence, and tumor understaging (83, 85).

Cystoscopy during TURBT has traditionally been performed using white light. A more recent alternative is blue light cystoscopy, also known as photodynamic diagnosis (PDD), which uses an intravesically administered photosensitizing agent that preferentially accumulates in tumor cells that can subsequently be detected from their fluorescence (86). Relative to white light cystoscopy (WLC), PDD has a higher sensitivity but lower specificity in bladder cancer detection (87), and the use of PDD may affect treatment decisions through improved detection of tumors (88). In guidelines, the use of PDD over WLC is currently a moderate recommendation in the AUA/SUO guideline (17) and a weak recommendation in the EAU guideline (11). Systematic reviews and meta-analyses have suggested that blue light cystoscopy is associated with reduced risks of disease recurrence and possibly disease progression relative to WLC. The benefits of PDD may depend on baseline risk and findings were uncertain given limitations in the available evidence base, including the differential use of adjuvant therapy (89–91).

These reviews did not include the recently published PHOTO RCT, which compared TURBT guided by PDD with standard WLC TURBT in 538 patients with NMIBC across centers in the UK (86). The trial found no evidence for differences in recurrence or progression risk and in overall or bladder cancer-specific survival between PDD-guided and standard WLC TURBT. At 3 years, PDD-guided TURBT was also unlikely to be cost-effective versus WLC TURBT. The authors concluded that PDD-guided TURBT could not be recommended for managing patients with primary intermediate- or high-risk NMIBC (86). The trial has generated some controversy, however, for its inconsistency with the existing literature (92). In particular, the trial has been criticized for excluding a substantial portion of randomly allocated patients from the analysis and for being underpowered to detect differences between PDD and WLC as its power calculations rested on risk reductions considered inadequate and as the planned patient accrual had not concluded by trial end. In addition, adjuvant therapy was not balanced between trial arms as higher rates of patients in the WLC arm received adjuvant therapy, including BCG. High-risk patients were also considered to be underrepresented in the trial (92). Given the association between (higher) baseline risk and (higher) benefit of PDD, the largely intermediate-risk group studied in PHOTO likely reduced the probability of detecting an effect of PDD (89). Differences in TURBT quality, influenced by surgeon experience, were also suggested as a potential reason why the trial failed to identify differences between PDD and WLC (93). Given these concerns, Stenzl et al. concluded that the PHOTO trial would not invalidate the available evidence in favor of PDD in NMIBC (92).

#### 2.4.2 Repeat TURBT

If the primary TURBT is incomplete or there is no detrusor muscle in the specimen, the second(-look) TURBT should be performed 4–6 weeks after the first resection (11, 17). AUA/Society of Urologic Oncology (SUO) guidelines recommend considering repeat TURBT also in high-risk Ta HG tumors and T1 tumors, while EAU guidelines consider only T1 tumors for repeat TURBT. Endorsement of re-resection in guidelines reflects consistent findings that second TURBT reduces recurrence risk and improves response to intravesical BCG in patients with high-risk or high-grade NMIBC (94–96). Initial evidence also suggests improved all-cause (but not cancer-specific) mortality associated with a second TURBT in patients with T1 bladder cancer (97). There appears to be no survival difference between re-TURBT performed before or after BCG induction (98).

#### 2.4.3 Single, immediate post-operative instillation of chemotherapy

Adjuvant treatment post-TURBT is recommended to start with a single, immediate instillation of chemotherapy, within 24 hours of surgery, optimally within 2 to 6 hours (11, 17, 99). Such a “single shot”, usually of MMC or gemcitabine, has been demonstrated to reduce recurrence risk in patients with NMIBC in all risk groups and regardless of adjuvant BCG therapy (78, 100, 101). The single post-TURBT instillation of chemotherapy is most effective in patients with low-grade Ta cancers (102). For low-risk patients, no treatment beyond this single immediate instillation is needed.

#### 2.4.4 Intravesical BCG

Subsequent adjuvant treatment in patients at intermediate and high risk is intravesical treatment with BCG or chemotherapy (11, 17, 99). Immunotherapy with BCG, a live attenuated strain of *Mycobacterium bovis*, is the mainstay intravesical treatment for NMIBC (7, 103). Clinical trials have shown that BCG is safe and effective in reducing tumor recurrence and progression (104–109). Multiple BCG strains are available, which are considered to have equivalent efficacy and safety (11, 17, 110–112).

Most guidelines recommend that BCG administration follows the so-called “6+3”, or SWOG, schedule (11, 17, 104, 113, 114). For high-risk patients, a 6-week induction period (instillations once a week) is followed by maintenance treatment of three instillations (one instillation per week over 3 weeks) at 3 and 6 months and then every 6 months thereafter, up to 36 months from initiation of induction therapy. For intermediate-risk patients, the same induction schedule is used, but maintenance is shortened to 1 year, with three instillations each given at 3, 6, and 12 months (11).

The SWOG protocol is not consistently applied in clinical practice and research, and many interventional studies consider only induction therapy or change the frequency, duration, or doses of BCG (115).

In particular, data on the benefit of maintenance therapy are inconsistent and often underpowered, and drawing definitive conclusions is therefore challenging (116). In the CUETO 98013 study, a maintenance schedule of one instillation every 3 months for 3 years was not associated with decreased recurrence or progression relative to induction-only therapy in high-risk patients (117). Palou et al. (118) found no recurrence or progression benefit of maintenance therapy given as six instillations every 6 months for 2 years relative to no maintenance therapy. In contrast, the SWOG 8507 study in high-risk patients showed clear benefits in RFS and worsening-free survival (defined as no evidence of progression) for the maintenance regimen now known as the SWOG schedule, compared to no maintenance therapy. The NIMBUS trial in high-grade NMIBC confirmed that a reduced BCG schedule is associated with increased recurrence risk versus the standard schedule (119). Similar results were reported in observational studies, although benefits for progression were not always shown (120, 121). Reduced recurrence and progression risks with maintenance BCG were shown in several systematic reviews and meta-analyses, regardless of patient risk group (122, 123); in turn, the superiority of BCG over MMC was found only if maintenance therapy was given (106, 107, 109).

On balance, maintenance therapy is likely beneficial, as stipulated by guidelines and consensus documents (11, 17, 99, 113). It appears plausible that between-study differences were influenced in part by different, regularly inadequate maintenance schedules. Inadequate dosing may also have contributed. While BCG doses are difficult to compare between studies as they are often reported by weight instead of colony-forming units, not all studies used the full doses recommended for treatment. In a randomized trial of intermediate- and high-risk patients, receiving one-third of a standard BCG dose was associated with shorter disease-free survival relative to a full dose, with no differences in toxicity (124). In high-risk patients, 3-year maintenance therapy decreased the recurrence risk with full, but not with reduced doses.

#### 2.4.5 Intravesical chemotherapy

Intravesical chemotherapy is an alternative to BCG in intermediate-risk NMIBC, in particular for recurrent low-grade disease. In high-risk patients, while BGC should be the preferred treatment option, chemohypothermia with mitomycin-C (MMC) is an alternative to BCG where BCG is unavailable or not tolerated (125–127). Pre-operative/neoadjuvant use of intravesical chemotherapy with MMC (also referred to as “chemoresection”) has also been suggested to help reduce the number of invasive procedures (TURBT) in patients with recurrent intermediate-risk NMIBC (128). In addition to MMC, other available chemotherapeutic agents include epirubicin, gemcitabine and docetaxel (129). Given the BCG shortage, sequential gemcitabine-docetaxel has been investigated as an alternative or salvage therapy in NMIBC. This sequential instillation treatment was found to be effective and well-tolerated in retrospective studies of high-risk patients who were BCG-naïve or had failed BCG (130, 131). A comparative retrospective study reported improved outcomes and reduced treatment discontinuation in treatment-naïve high-risk patients for gemcitabine-docetaxel relative to BCG (132). However, the comparison of contemporary gemcitabine-docetaxel patients with a historic BCG cohort, with different follow-up periods and the use of a non-standard reduced-dose BCG-IFN-2α combination treatment as comparator, limit the interpretation of these results. A recent systematic review of sequential gemcitabine-docetaxel therapy concluded that the therapy was promising, but that additional large studies of good quality that compare gemcitabine-docetaxel with BCG are necessary (133).

BCG shortages have forced urologists to investigate strategies to improve the efficacy of chemotherapy delivery. The concept of device-assisted intravesical therapy, particularly chemohyperthermia (hyperthermic intravesical chemotherapy [HIVEC] or radiofrequency-induced thermo-chemotherapy effect [RITE]), to improve the penetration of MMC and other chemotherapeutic agents into the bladder wall appears promising as enhanced antitumor effects have been evident. However, despite accumulating data in the BCG-naïve (134–137) and BCG-unresponsive settings (138, 139), intravesical device-assisted chemotherapy is not broadly available for NMIBC patients (134–136, 140–145). Electromotive Drug Administration (EMDA) improves penetration of chemotherapy agents by electroosmosis, iontophoresis, and electroporation (140) but as with chemohyperthermia, is not yet recommended in treatment guidelines due to insufficient evidence (140, 146).

#### 2.4.6 Radical cystectomy

Radical cystectomy is the gold standard treatment for MIBC, and early radical cystectomy is the preferred treatment in patients with very high-risk NMIBC, although recent evidence also suggests a clinical benefit of intravesical BCG in these patients (11, 125, 147). Early radical cystectomy is also recommended for BCG-unresponsive disease (11). While the high-risk characteristics prompting eligibility for radical cystectomy differ between guidelines (**Supplementary Tables S1**, **S2**), studies evaluating the benefits of early radical cystectomy have not differentiated BCG-naïve patients from those who have failed BCG. The benefit of early radical cystectomy in BCG-naïve patients, therefore, remains unclear. The BRAVO-Feasibility study demonstrated that patients with high-grade NMIBC treated with early radical cystectomy experience acceptable outcomes, but the feasibility of recruiting into a larger trial was considered doubtful, particularly as the majority of patients preferred bladder preservation (148).

#### 2.4.7 Novel pharmaceutical agents

Novel pharmaceutical agents as well as improvements to existing intravesical therapy for second-line treatment of NMIBC are under investigation (149, 150). Agents under investigation span multiple modalities and therapeutic classes, including chemohyperthermia with MMC, recombinant viruses (IFNα2b-encoding nadofaragene firadenovec), oncolytic viruses (GM-CSF-encoding adenovirus CG0070), immune checkpoint inhibitors (ICIs; PD-1 and PD-L1 inhibitors such as pembrolizumab, cetrelimab, atezolizumab, durvalumab and sasanlimab), immunomodulatory agents (IL-15 superagonist nogapendekin alfa inbakicept [NAI] or N-803), photodynamic therapy (PDT; ruthenium-based photosensitizer TLD1433), a Nectin-4-directed antibody and microtubule inhibitor conjugate (enfortumab vedotin), and a pan-FGFR tyrosine kinase inhibitor (erdafitinib). Furthermore, many of these agents are currently being investigated in combination, including N-803 in combination with BCG, ICIs in combination with BCG, CG0070 in combination with pembrolizumab, and cetrelimab in combination with gemcitabine (151). New agents that are available as intravesical formulations are generally favored by urologists.

Among immunotherapies, pembrolizumab has been approved by the US Food and Drug Administration (FDA) for the treatment of BCG-unresponsive, high-risk NMIBC with Cis with or without papillary tumors. In December 2022, the FDA also approved nadofaragene firadenovec, a gene therapy using a recombinant adenovirus as its vector, for the same indication, but this option is not yet clinically available. As of writing, pembrolizumab and nadofaragene firadenovec have not been approved for the treatment of NMIBC by the European Medicines Agency (EMA). An FDA decision on the use of N-803 in combination with BCG is due in May 2023.

## 3 Discussion

Despite the approval of novel and the effectiveness of already available treatments, recurrence and, in high-risk disease, progression rates remain high. In many instances, treatment failure can be attributed to non-adherence to guideline recommendations across the treatment pathway, from TURBT to intravesical therapy and early radical cystectomy. Although guidelines differ to some extent and vary in quality (82, 125), recommendations for the treatment of high-risk NMIBC are sufficiently aligned and based on evidence of sufficient quality to warrant familiarity and consistent use of guidelines.

Examples of guideline non-adherence include not administering repeat TURBT in high-grade T1 NMIBC and possibly high-grade Ta NMIBC if the initial TURBT failed to retrieve detrusor muscle, not using single immediate instillations of chemotherapy following TURBT, delaying BCG therapy, and not administering BCG maintenance therapy or full BCG doses to achieve the best possible outcomes (3, 5, 85, 119, 124, 152–155). In a systematic review, Mori et al. (156) evaluated compliance rates for most steps in a typical NMIBC treatment pathway. They reported overall compliance rates between 33%, for the administration of BCG in high-risk NMIBC, to 82% for follow-up cystoscopy. Repeat TURBT was performed in fewer than half of high-risk patients (43%), and only about a third (37%) of intermediate-risk patients received intravesical therapy. Studies published since then continue to show treatment to be underused, in particular single, immediate chemotherapy instillations, second TURBT, and BCG maintenance (**Supplementary Table S3**) – fewer than a third of US patients with T1 disease received guideline-based treatment between 2004–2014 (157, 158).

The reasons for this unmet need can be categorized as lack of knowledge and training [or adherence to “myths and mysteries” concerning BCG (114)], lack of access, economic barriers, fear of side effects, and patient comorbidities (**Supplementary Table S4**). Adequate training and knowledge are important determinants of guideline-compliant treatment, e.g., for use of single, immediate instillations of chemotherapy (159). Second TURBT and intravesical therapy were more likely if treatment was performed in academic institutions and by a specialist (157, 158, 160, 161). Urologists with less time in practice were more likely to treat in compliance with guidelines than those with more experience, potentially because younger physicians were more willing to consult guidelines to compensate for their (perceived) lack of experience (157, 160).

Economic barriers have also been reported, for instance for uninsured patients in the US and for Chinese patients who refused indicated surgeries and treatment due to financial concerns (158, 162). The shortage of BCG (discussed below), is another reason why treatment, in particular BCG maintenance therapy, may not be performed in compliance with guidelines (160, 162). Concerns about side effects and patient comorbidities represent further causes of non-guideline-compliant treatment. Between 20–60% of patients do not receive second TURBT or intravesical therapy due to their general health (161). Such concerns may be voiced by treating physicians, but also by patients worrying about potential side effects and reduced QoL (162).

Guideline-based treatment has become more common (142, 148), but additional efforts are needed to make it the default. Suggestions discussed in the literature include, firstly, improvements to risk stratification models. More reliable models would help to facilitate targeted and personalized treatment decisions, including early identification of patients unlikely to benefit from specific treatments such as BCG (163–165). Secondly, guideline adherence could be made more prominent in clinical practice by defining quality performance indicators (QPI) that include key guideline recommendations. Perhaps the best-known example of such an approach and its success is the Scottish QPI Programme, which developed and implemented bladder cancer-related QPIs in Scotland from 2012 onwards, with clinical outcomes being evaluated through the Scot BC Quality OPS clinical project (166). The project has resulted in improved TURBT quality (including using single, immediate instillations of chemotherapy) with subsequent long-term recurrence benefits (152, 154). These indicators, if audited and reviewed with clinical staff, have increased motivation not to underperform compared to peers. Modern instillation technology and relatively simple changes such as including reminders in surgical documentation also contributed to improved care quality (159). Thirdly, guidelines and their contents as well as QPIs could be made more accessible using modern information technology. Beardo et al. (167) have presented initial results from using a computer application that combines clinicopathological information with treatment suggestions derived from guidelines. Initial results were promising, with reduced rates of recurrence in cases where the app- and urologist-proposed treatments agree, relative to cases without agreement. Further validation work is needed, but such an application may aid physicians in treatment decision-making. Fourthly, for the very heterogenous group of intermediate-risk NMIBC, further efforts into improved risk substratification, research into the relative benefits of chemotherapy versus immunotherapy, and optimized intravesical chemotherapy schedules, and treatment harmonization are required, such as recent work by the International Bladder Cancer Group (137).

Apart from suboptimal treatment, the outcomes of NMIBC are negatively affected by insufficient patient lifestyle changes, including smoking cessation (63, 168–171). Adherence to lifestyle recommendations is associated with reduced recurrence risk and improved QoL in patients with NMIBC (172, 173), but several surveys have shown low rates of lifestyle changes following NMIBC diagnosis (**Table 1**). Among Dutch patients, only 36% of smokers had stopped smoking 3 months after the diagnosis (171). A recent analysis of UroLife data confirmed largely unchanged lifestyles following NMIBC diagnosis – only minor to moderate changes were found for most lifestyle categories, most smokers continued to smoke, and BMI remained unchanged in the first 15 months since diagnosis (168). For the UK, an even lower smoking cessation rate of 14% was reported over 4.2 years after diagnosis (169). In Turkish patients, a higher cessation rate (58%) was reported (170). This lack of adherence to lifestyle recommendations likely results from patients being insufficiently aware of risk factors and detrimental lifestyle. Among Dutch patients, most (89%) identified smoking as a cancer risk factor but only between 29% and 67% were knowledgeable about other risk factors such as insufficient consumption of vegetables and fruit or being overweight (171).

**Table 1:** Lifestyle changes in patients with NMIBC.

Study, study design	Findings on patient lifestyle changes
Beeren et al. (168), survey of Dutch patients from the UroLife study (n=966: smoking, n=961: physical activity, n=960: BMI, n=949: diet, n=935: overall WCRF/AICR recommendation score)	Small to moderate albeit statistically significant effect sizes for lifestyle change (for all domains except BMI), baseline → 15 months: • Overall WCRF/AICR: 3.3 → 3.3 • BMI: 26.8 → 26.9 kg/m^2^ • Physical activity: 750 → 665 min/week • Red and processed meat: 704 → 608 g/week • Smoking: 23.2 → 15.0%
Van Osch et al. (169), semi-structured face-to-face interviews and questionnaires in 722 UK patients with NMIBC at risk of recurrence	• Of 174 patients smoking at diagnosis, 25 (14%) quit over a median of 4.21 years of follow-up • No statistically significant effect of smoking cessation on 5-year recurrence risk but too few cessation events for firm conclusions
Westhoff et al. (171), survey of 969 Dutch patients with NMIBC from the UroLife cohort	• Patients receiving lifestyle advice from a physician on: o Smoking cessation (in smokers): 70% o Weight loss (in patients with BMI >25 kg/m^2^): 6% (34% considered that weight loss advice did not apply to them) o Healthy diet: 9% o Physical activity: 15% • Smoking cessation at 3 months after diagnosis: 36%
Yuruk et al. (170), survey of 187 Turkish patients with NMIBC enrolled under cystoscopic surveillance	• At diagnosis, 61% of patients were active smokers, of whom 76% received advice to quit smoking and of whom 58% quit smoking • 4.1% of current or ex-smokers had been referred to smoking cessation programs

AICR, American Institute for Cancer Research; NMIBC, Non-Muscle-Invasive Bladder Cancer; UK, United Kingdom; WCRF, World Cancer Research Fund.

Patients may not receive enough counseling and assistance from healthcare providers. Only 29% of current smokers that had been advised to quit smoking had received an offer of help to stop smoking in the Netherlands, and only 21% of all patients reported advice from their physician on at least one lifestyle behavior (171). In Turkey, only 4.1% of current or ex-smokers have been referred to a smoking cessation program (170). Further evidence on the effectiveness and practicality of lifestyle recommendations would also be required to increase awareness of and confidence in such recommendations (172).

Patients may not only find it difficult to adhere to lifestyle recommendations – their compliance with surveillance and treatment regimens is also often suboptimal, leading to frequent treatment discontinuation and less favorable clinical outcomes (5, 124, 161, 174–179).

NMIBC care is beset by high rates of treatment discontinuation, particularly for intravesical therapy (**Supplementary Table S5**). Compliance with cystoscopic surveillance protocols has been reported at below 20% (174, 179). For intravesical BCG, completion of induction therapy is 80–90%, but completion of maintenance therapy varies between 10–76% – depending in particular on the proposed maintenance duration since longer therapy was less likely to be completed (19, 124, 161, 175–177). Side effects of BCG therapy, particularly in the first year of treatment, may contribute to treatment discontinuation, with proportions of patients stopping BCG treatment in trials reported between 8% and 20% (177, 178, 180, 181). Not complying with treatment and surveillance protocols was associated with increased rates of clinical events, including recurrence, progression, and mortality, of which patients may not be sufficiently aware when deciding to discontinue treatment (174, 175, 179) or surveillance (179).

Toxicities and adverse events are important reasons why patients discontinue or do not start treatment, particularly for BCG and radical cystectomy (19, 176, 178). Although BCG is generally safe, and most associated toxicities and adverse events are transient, patients may be deterred from continuing with therapy, especially if activities of daily living and social activities are impaired (176, 178, 182). The BCG shortage is another reason for not starting or continuing (maintenance) therapy (175, 176). The shortage was triggered by the closure of a BCG-producing facility and subsequent struggles by other manufacturers to meet the production shortfall. The BCG shortage has negatively affected timely treatment and patient outcomes, and has increased treatment costs in some settings (76, 183–186). A more recent interruption to treatment for many patients was the COVID-19 pandemic. In studies from Italy, Turkey, and the US, delays to second resection, lower rates of maintenance therapy, and increased risks of missed treatments were reported (187, 188). In the Netherlands, bladder cancer diagnoses decreased during the first wave of the pandemic, but care was less affected than for other cancers and in other countries (189).

Potential solutions to high treatment discontinuation rates will likely have to include further enhancements to BCG tolerability, with improved prevention and management of BCG side effects (190). Communication between physicians and patients on side effects, including risk, severity, and meaning (e.g., inflammation as a painful but promising sign in line with BCG’s expected mechanism), might improve compliance and would likely be met with interest from patients (190, 191). Similarly, physicians should not assume patient awareness of risk factors or beneficial lifestyle changes. Instead, healthcare staff should aim, within the acknowledged constraints of a busy schedule, to inform and advise patients on the disease and how patients could adapt their lifestyles (171). It should be noted, however, that not all patients appreciate lifestyle advice – among Dutch patients, 80% thought lifestyle advice was beneficial and 70% agreed that physicians had a duty to provide advice, but 15% perceived advice as intrusive and superfluous (171).

The BCG shortage is more difficult to address. Manufacturers have announced production increases, but given the complexity of BCG manufacturing, supply increases will take time (192). Until BCG supply meets demand, alternative treatments and modified BCG treatment approaches could be explored and available resources more carefully used, although treatment schedules and doses should remain guideline-based as far as possible (11, 193). While future developments of the COVID-19 pandemic remain uncertain, the first waves can be considered a chance to improve bladder cancer and NMIBC care, by highlighting areas worthy of (re-)prioritization. This may include avoidance of overtreatment and excessive surveillance in patients at low risk (166, 194) and an increased focus on timely diagnosis, TURBT, and adjuvant therapy initiation (195, 196).

The unmet needs discussed so far are mostly a consequence of physician and patient behavior. They are accompanied by evidence gaps that make informed treatment decisions challenging. In particular, a corollary of suboptimal, non-guideline-compliant treatment and different treatment schedules is the limited ability to compare outcomes across studies and draw conclusions about relative effectiveness and safety, including on the optimal management of side effects of BCG (197–200). Differences in BCG administration schedules and dosing have made it challenging to compare efficacy and safety outcomes between studies (201). It remains difficult to establish if BCG therapy failure is a genuine failure of BCG or reflects inadequate treatment. The challenge of having to combine estimates from studies using different intravesical schedules affects several systematic reviews and meta-analyses. As an example, a review comparing BCG with MMC for Ta and T1 bladder cancer included, among others, a study using 6-week induction of MMC (two arms with the same dose but different instillation times) and BCG, followed by ten once-monthly instillations (if no recurrence) or second induction for 6 weeks (if recurrence); a study using six weekly instillations for BCG and MCC and with one arm of monthly MMC for another 3 years after induction; a study comparing MMC given every 2 weeks in the first and then every 4 weeks in the second year with BCG given weekly over 6 weeks and then once a month for 4 months (127). Other reviews face similar challenges with heterogeneous data (109, 126).

These concerns have led to the definition of “adequate BCG therapy” when defining “BCG-unresponsive” disease. For clinical trials, adequate therapy has been defined as ≥5 of six induction and ≥2 of three maintenance doses or as ≥5 of six induction and ≥2 of six second induction courses (32, 33). Defining adequate BCG therapy provides some standardization of BCG schedules that improve between-study comparability, and the definition has already seen use in published studies (3, 202). The definition also allows for defining BCG-unresponsive disease, i.e., distinguishing insufficient BCG administration from cases of BCG failure, in clinical trial design (11). Outside clinical trials, up to 21% of patients have been reported as BCG-unresponsive based on data collected mainly in the 2010s, and unresponsive patients have significantly reduced overall and cancer-specific survival relative to responsive patients (203). More recent data have pointed to improved outcomes with adequate BCG in high-risk patients (119, 204), in patients with Cis (3, 202), and even in patients with very high risk disease (22, 204, 205).

If these definitions and concepts are consistently applied in clinical practice, they should help facilitate study conduct and evidence synthesis in the future. The increasing availability of high-quality evidence, e.g., from clinical trials instead of observational studies (119), should also contribute to the accuracy and robustness of evidence synthesis. For intermediate-risk NMIBC, further work is required on identifying optimal therapies, including the benefits of immune- versus chemotherapy and of maintenance therapy as recurrence rates in this patient group remain unsatisfactorily high (5, 126, 136, 137, 206–210). In high-risk patients, data on the time to initiation of BCG and on the effects of delays in BCG initiation remain sparse, and further data would be welcome (155).

The quality of clinical trials in patients with NMIBC also suffers from inconsistent outcome reporting and under-representation of certain patient groups. In a review of fifty-seven randomized controlled trials, Veskimae et al. (211) found that, while recurrence, progression, treatment response, and adverse events were reported in most studies. Overall and cancer-specific survival, treatment discontinuation, QoL, and economic outcomes were less frequently or rarely reported. The review further found substantial variation in how recurrence and progression were labeled and defined; for example, progression was defined as progression to MIBC, with or without including metastases, or as patients moving from Ta to T1 and from T1 to MIBC.

Trial populations also often do not represent real-world patient collectives. A review of phase 1–3 studies from the US and Canada on BCG-unresponsive disease found that only 41% of trials reported data on race/ethnicity. Trials that reported race/ethnicity mostly included white participants (94%), which did not reflect the burden of bladder cancer in racial/ethnic minorities (212). These findings were mirrored when comparing cancer incidence proportions with trial population proportions in US studies, which showed Black and Hispanic patients to be underrepresented in bladder cancer trials relative to non-Hispanic white patients (213). The proportion of Black patients enrolled in trials remained unchanged between 2000 and 2019.

More stringent definitions of treatment schedules and ideally a core outcome set as well as design guidelines for bladder cancer trials should be able to reduce this unmet evidence need (32, 33, 211, 214). Increased patient involvement in trial design could increase the likelihood that patient-relevant outcomes are considered (73). An increased commitment to diversity and inclusivity among trial sponsors and investigators to combat under-representations is required (215).

Not only the available clinical data, but also the available scoring models and risk stratification tables are often limited in their usefulness, which increases the difficulty of selecting the right treatment for patients. Validation studies are available for several prominent models (**Supplementary Table S6**). For all, validation exercises show reduced discriminative ability and differences in estimated risks if applied to different populations, although it must be acknowledged that not all cohorts used for validation work reflect the current standard of care and improved treatment (29, 92). In a recent validation study of the new EAU 2021 model, for example, the model’s discriminative ability was found to be reduced, and progression risks were overestimated, when used in US patients treated with BCG (22). Risk stratification and subsequent treatment selection are especially challenging for patients with intermediate-risk NMIBC, given the clinically heterogeneous makeup of this patient group (137).

These issues should be addressed by continuous validation of existing models in different populations, including in non-European and non-US cohorts, should be performed to further assess which model is suitable in which populations. In addition, model developers should clearly communicate potential model shortcomings. Models can likely be further improved by applying novel statistical and computational tools and by considering additional predictors as well as using ever larger datasets (216, 217). There is also likely untapped potential in biomarkers as predictors of recurrence and progression risk (20, 164, 192, 218).

## 4 Conclusion

This study presented an overview of NMIBC, including its burden and treatment options, and summarized current unmet needs in NMIBC care and evidence generation. There are many aspects of treatment that are not standardized, including the quality of TURBT, repeat TURBT and intravesical therapy with BCG or chemotherapy. Standardization efforts, like the definition of BCG-unresponsive disease and “adequate” BCG therapy, are new, and guideline recommendations are often not followed in clinical practice and interventional studies. This not only needlessly risks suboptimal patient outcomes in a disease for which effective and safe treatments have been available for decades, but also limits study comparability and evidence synthesis. Addressing these unmet needs should be a priority in clinical practice and research. The present review offered some suggestions as to how these needs could be addressed, including increasing treatment guideline awareness among physicians through improved training, technological advances, and the use of QPIs; strengthening the focus on optimal prevention and management of side effects of intravesical treatment; and changing the mindset about the biology of bladder cancer as a disease of “field cancerization” requiring field rather than local treatment.

## Author contributions

KG-H formulated the idea for the present review. Literature searches were performed by RP and JP. The first draft of the manuscript was written by JP. KG-H, CH, PM, GG, and RP commented on and revised earlier versions of the manuscript. KG-H, CH, PM, GG, RP, and JP contributed to the study’s design and/or data analysis and interpretation. KG-H, CH, PM, GG, RP, and JP read and approved the final manuscript. JP, RP, and CH prepared responses to peer review comments. All authors contributed to the article and approved the submitted version.

## References

[1] Richters A Aben KKH Kiemeney LALM. The global burden of urinary bladder cancer: an update. World J Urol (2020) 38:1895–904. doi: 10.1007/s00345-019-02984-4 PMC7363726 31676912

[2] Safiri S Kolahi A-A Naghavi M Global Burden of Disease Bladder Cancer Collaborators. Global, regional and national burden of bladder cancer and its attributable risk factors in 204 countries and territories, 1990-2019: a systematic analysis for the global burden of disease study 2019. BMJ Glob Health (2021) 6:e004128. doi: 10.1136/bmjgh-2020-004128 PMC8634015 34844997

[3] Matulay JT Li R Hensley PJ Brooks NA Narayan VM Grossman HB. Contemporary outcomes of patients with nonmuscle-invasive bladder cancer treated with bacillus calmette-guérin: implications for clinical trial design. J Urol (2021) 205:1612–21. doi: 10.1097/JU.0000000000001633 33502236

[4] Dovey Z Pfail J Martini A Steineck G Dey L Renström L. Bladder cancer (NMIBC) in a population-based cohort from Stockholm county with long-term follow-up; a comparative analysis of prediction models for recurrence and progression, including external validation of the updated 2021 E.A.U. model. Urol Oncol (2022) 40:106.e1–106.e10. doi: 10.1016/j.urolonc.2021.10.008 34840075

[5] Musat MG Kwon CS Masters E Sikirica S Pijush DB Forsythe A. Treatment outcomes of high-risk non-muscle invasive bladder cancer (HR-NMIBC) in real-world evidence (RWE) studies: systematic literature review (SLR). Clin Outcomes Res (2022) 14:35–48. doi: 10.2147/CEOR.S341896 PMC8759992 35046678

[6] Pettenati C Ingersoll MA. Mechanisms of BCG immunotherapy and its outlook for bladder cancer. Nat Rev Urol (2018) 15:615–25. doi: 10.1038/s41585-018-0055-4 29991725

[7] Lobo N Brooks NA Zlotta AR Cirillo JD Boorjian S Black PC. 100 years of bacillus calmette–guérin immunotherapy: from cattle to COVID-19. Nat Rev Urol (2021) 18:611–22. doi: 10.1038/s41585-021-00481-1 PMC8204595 34131332

[8] Teoh JY-C Huang J Ko WY-K Lok V Choi P Ng C-F. Global trends of bladder cancer incidence and mortality, and their associations with tobacco use and gross domestic product per capita. Eur Urol (2020) 78:893–906. doi: 10.1016/j.eururo.2020.09.006 32972792

[9] van Hoogstraten LMC Vrieling A van der Heijden AG Kogevinas M Richters A Kiemeney LA. Global trends in the epidemiology of bladder cancer: challenges for public health and clinical practice. Nat Rev Clin Oncol (2023) 20:287–304. doi: 10.1038/s41571-023-00744-3 36914746

[10] Magers MJ Lopez-Beltran A Montironi R Williamson SR Kaimakliotis HZ Cheng L. Staging of bladder cancer. Histopathology (2019) 74:112–34. doi: 10.1111/his.13734 30565300

[11] Babjuk M Burger M Capoun O Cohen D Compérat EM Dominguez Escrig JL. European Association of urology guidelines on non-muscle-invasive bladder cancer (Ta, T1, and carcinoma *in situ*). Eur Urol (2022) 81:75–94. doi: 10.1016/j.eururo.2021.08.010 34511303

[12] Compérat EM Burger M Gontero P Mostafid AH Palou J Rouprêt M. Grading of urothelial carcinoma and the new “World health organisation classification of tumours of the urinary system and Male genital organs 2016”. Eur Urol Focus (2019) 5:457–66. doi: 10.1016/j.euf.2018.01.003 29366854

[13] Humphrey PA Moch H Cubilla AL Ulbright TM Reuter VE. The 2016 WHO classification of tumours of the urinary system and Male genital organs–part b: prostate and bladder tumours. Eur Urol (2016) 70:106–19. doi: 10.1016/j.eururo.2016.02.028 26996659

[14] van Rhijn BWG Hentschel AE Bründl J Compérat EM Hernández V Čapoun O. Prognostic value of the WHO 1973 and WHO 2004/2016 classification systems for grade in primary ta/t1 non-muscle-invasive bladder cancer: a multicenter European association of urology non-Muscle-Invasive bladder cancer guidelines panel study. Eur Urol Oncol (2021) 4:182–91. doi: 10.1016/j.euo.2020.12.002 33423944

[15] Fernandez-Gomez J Madero R Solsona E Unda M Martinez-Piñeiro L Gonzalez M. Predicting nonmuscle invasive bladder cancer recurrence and progression in patients treated with bacillus calmette-guerin: the CUETO scoring model. J Urol (2009) 182:2195–203. doi: 10.1016/j.juro.2009.07.016 19758621

[16] Sylvester RJ Rodríguez O Hernández V Turturica D Bauerová L Bruins HM. European Association of urology (EAU) prognostic factor risk groups for non–muscle-invasive bladder cancer (NMIBC) incorporating the WHO 2004/2016 and WHO 1973 classification systems for grade: an update from the EAU NMIBC guidelines panel. Eur Urol (2021) 79:480–8. doi: 10.1016/j.eururo.2020.12.033 33419683

[17] Chang SS Boorjian SA Chou R Clark PE Daneshmand S Konety BR. Diagnosis and treatment of non-muscle invasive bladder cancer: AUA/SUO guideline, 2016; amended 2020 (2020). Available at: https://www.auanet.org/documents/education/clinical-guidance/Non-Muscle-Invasive-Bladder-Cancer.pdf (Accessed February 3, 2023).

[18] Sylvester RJ van der Meijden APM Oosterlinck W Witjes JA Bouffioux C Denis L. Predicting recurrence and progression in individual patients with stage Ta T1 bladder cancer using EORTC risk tables: a combined analysis of 2596 patients from seven EORTC trials. Eur Urol (2006) 49:466–5. doi: 10.1016/j.eururo.2005.12.031 16442208

[19] Gontero P Sylvester R Pisano F Joniau S Vander Eeckt K Serretta V. Prognostic factors and risk groups in T1G3 non-muscle-invasive bladder cancer patients initially treated with bacillus calmette-guérin: results of a retrospective multicenter study of 2451 patients. Eur Urol (2015) 67:74–82. doi: 10.1016/j.eururo.2014.06.040 25043942

[20] Dyrskjøt L Reinert T Algaba F Christensen E Nieboer D Hermann GG. Prognostic impact of a 12-gene progression score in non-muscle-invasive bladder cancer: a prospective multicentre validation study. Eur Urol (2017) 72:461–9. doi: 10.1016/j.eururo.2017.05.040 28583312

[21] Ferro M Di Mauro M Cimino S Morgia G Lucarelli G Abu Farhan AR. Systemic combining inflammatory score (SCIS): a new score for prediction of oncologic outcomes in patients with high-risk non-muscle-invasive urothelial bladder cancer. Transl Androl Urol (2021) 10:626–35. doi: 10.21037/tau-20-1272 PMC7947442 33718065

[22] Lobo N Hensley PJ Bree KK Nogueras-Gonzalez GM Navai N Dinney CP. Updated European association of urology (EAU) prognostic factor risk groups overestimate the risk of progression in patients with non-muscle-invasive bladder cancer treated with bacillus calmette-guérin. Eur Urol Oncol (2022) 5:84–91. doi: 10.1016/j.euo.2021.11.006 34920986 PMC11902298

[23] Krajewski W Aumatell J Subiela JD Nowak Ł Tukiendorf A Moschini M. Accuracy of the CUETO, EORTC 2016 and EAU 2021 scoring models and risk stratification tables to predict outcomes in high-grade non-muscle-invasive urothelial bladder cancer. Urol Oncol (2022) 40:491.e11–491.e19. doi: 10.1016/j.urolonc.2022.06.008 35851185

[24] Krajewski W Rodríguez-Faba O Breda A Pisano F Poletajew S Tukiendorf A. Validation of the CUETO scoring model for predicting recurrence and progression in T1G3 urothelial carcinoma of the bladder. Actas Urol Esp (2019) 43:445–51. doi: 10.1016/j.acuro.2019.02.006 31155372

[25] Rosevear HM Lightfoot AJ Nepple KG O’Donnell MA. Usefulness of the Spanish urological club for oncological treatment scoring model to predict nonmuscle invasive bladder cancer recurrence in patients treated with intravesical bacillus calmette-guérin plus interferon-α. J Urol (2011) 185:67–71. doi: 10.1016/j.juro.2010.08.083 21074202

[26] Jobczyk M Stawiski K Fendler W Różański W. Validation of EORTC, CUETO, and EAU risk stratification in prediction of recurrence, progression, and death of patients with initially non–muscle-invasive bladder cancer (NMIBC): a cohort analysis. Cancer Med (2020) 9:4014–25. doi: 10.1002/cam4.3007 PMC7286464 32216043

[27] Ritch CR Velasquez MC Kwon D Becerra MF Soodana-Prakash N Atluri VS. Use and validation of the AUA/SUO risk grouping for nonmuscle invasive bladder cancer in a contemporary cohort. J Urol (2020) 203:505–11. doi: 10.1097/JU.0000000000000593 31609178

[28] Soria F D’Andrea D Abufaraj M Moschini M Giordano A Gust KM. Stratification of intermediate-risk non-muscle-invasive bladder cancer patients: implications for adjuvant therapies. Eur Urol Focus (2021) 7:566–73. doi: 10.1016/j.euf.2020.05.004 32532704

[29] Contieri R Hurle R Paciotti M Casale P Saita A Porpiglia F. Accuracy of the European association of urology (EAU) NMIBC 2021 scoring model in predicting progression in a large cohort of HG T1 NMIBC patients treated with BCG. Minerva Urol Nephrol (2023) 75:180–7. doi: 10.23736/S2724-6051.22.04953-9 36197700

[30] Fernandez-Gomez J Madero R Solsona E Unda M Martinez-Piñeiro L Ojea A. The EORTC tables overestimate the risk of recurrence and progression in patients with non-muscle-invasive bladder cancer treated with bacillus calmette-guérin: external validation of the EORTC risk tables. Eur Urol (2011) 60:423–30. doi: 10.1016/j.eururo.2011.05.033 21621906

[31] Cambier S Sylvester RJ Collette L Gontero P Brausi MA van Andel G. EORTC nomograms and risk groups for predicting recurrence, progression, and disease-specific and overall survival in non–muscle-invasive stage Ta–T1 urothelial bladder cancer patients treated with 1–3 years of maintenance bacillus calmette-guérin. Eur Urol (2016) 69:60–9. doi: 10.1016/j.eururo.2015.06.045 26210894

[32] Kamat AM Sylvester RJ Böhle A Palou J Lamm DL Brausi M. Definitions, end points, and clinical trial designs for non-muscle-invasive bladder cancer: recommendations from the international bladder cancer group. J Clin Oncol (2016) 34:1935–44. doi: 10.1200/JCO.2015.64.4070 PMC5321095 26811532

[33] Food and Drug Administration. BCG-Unresponsive nonmuscle invasive bladder cancer: developing drugs and biologics for treatment: guidance for industry (2018). Silver Spring, MD: US Department of Health and Human Services Food and Drug Administration. Available at: https://www.fda.gov/media/101468/download (Accessed February 3, 2023).

[34] Williams SB Howard LE Foster ML Klaassen Z Sieluk J De Hoedt AM. Estimated costs and long-term outcomes of patients with high-risk non–muscle-invasive bladder cancer treated with bacillus calmette-guérin in the veterans affairs health system. JAMA Netw Open (2021) 4:e213800. doi: 10.1001/jamanetworkopen.2021.3800 33787908 PMC8013821

[35] Seo M Langabeer JR II. Demographic and survivorship disparities in non–muscle-invasive bladder cancer in the united states. J Prev Med Pub Health (2018) 51:242–7. doi: 10.3961/jpmph.18.092 PMC6182276 30286596

[36] Wang EYH Pihlström N Malmström P-U Gårdmark T. Improved long-term outcome of patients with non-muscle invasive, low and intermediate risk bladder cancer between 1997 and 2014; a Swedish population-based study. Scand J Urol (2022) 56:221–6. doi: 10.1080/21681805.2022.2062046 35575423

[37] Kamat AM Hahn NM Efstathiou JA Lerner SP Malmström P-U Choi W. Bladder cancer. Lancet (2016) 388:2796–810. doi: 10.1016/S0140-6736(16)30512-8 27345655

[38] Sung H Ferlay J Siegel RL Laversanne M Soerjomataram I Jemal A. Global cancer statistics 2020: GLOBOCAN estimates of incidence and mortality worldwide for 36 cancers in 185 countries. CA Cancer J Clin (2021) 71:209–49. doi: 10.3322/caac.21660 33538338

[39] Shariat SF Sfakianos JP Droller MJ Karakiewicz PI Meryn S Bochner BH. The effect of age and gender on bladder cancer: a critical review of the literature. BJU Int (2010) 105:300–8. doi: 10.1111/j.1464-410X.2009.09076.x PMC4315315 19912200

[40] Schultzel M Saltzstein SL Downs TM Shimasaki S Sanders C Sadler GR. Late age (85 years or older) peak incidence of bladder cancer. J Urol (2008) 179:1302–1305; discussion 1305-1306. doi: 10.1016/j.juro.2007.11.079 18289593 PMC3751586

[41] Dobruch J Daneshmand S Fisch M Lotan Y Noon AP Resnick MJ. Gender and bladder cancer: a collaborative review of etiology, biology, and outcomes. Eur Urol (2016) 69:300–10. doi: 10.1016/j.eururo.2015.08.037 26346676

[42] Bilski K Zapała Ł Skrzypczyk MA Oszczudłowski M Dobruch J. Review on gender differences in non-muscle invasive bladder cancer. Transl Androl Urol (2019) 8:12–20. doi: 10.21037/tau.2018.11.06 30976563 PMC6414341

[43] Richters A Leliveld AM Goossens-Laan CA Aben KKH Özdemir BC. Sex differences in treatment patterns for non-advanced muscle-invasive bladder cancer: a descriptive analysis of 3484 patients of the Netherlands cancer registry. World J Urol (2022) 40:2275–81. doi: 10.1007/s00345-022-04080-6 PMC9427875 35778577

[44] Cumberbatch MGK Jubber I Black PC Esperto F Figueroa JD Kamat AM. Epidemiology of bladder cancer: a systematic review and contemporary update of risk factors in 2018. Eur Urol (2018) 74:784–95. doi: 10.1016/j.eururo.2018.09.001 30268659

[45] van Osch FHM Jochems SH van Schooten F-J Bryan RT Zeegers MP. Quantified relations between exposure to tobacco smoking and bladder cancer risk: a meta-analysis of 89 observational studies. Int J Epidemiol (2016) 45:857–70. doi: 10.1093/ije/dyw044 27097748

[46] Yan H Ying Y Xie H Li J Wang X He L. Secondhand smoking increases bladder cancer risk in nonsmoking population: a meta-analysis. Cancer Manag Res (2018) 10:3781–91. doi: 10.2147/CMAR.S175062 PMC6159806 30288109

[47] Kwan ML Haque R Young-Wolff KC Lee VS Roh JM Ergas IJ. Smoking behaviors and prognosis in patients with non-muscle-invasive bladder cancer in the be-well study. JAMA Netw Open (2022) 5:e2244430. doi: 10.1001/jamanetworkopen.2022.44430 36449286 PMC9713602

[48] Zuniga KB Graff RE Feiger DB Meng MV Porten SP Kenfield SA. Lifestyle and non-muscle invasive bladder cancer recurrence, progression, and mortality: available research and future directions. Bladder Cancer (2020) 6:9–23. doi: 10.3233/blc-190249 34095407 PMC8174672

[49] Crippa A Larsson SC Discacciati A Wolk A Orsini N. Red and processed meat consumption and risk of bladder cancer: a dose-response meta-analysis of epidemiological studies. Eur J Nutr (2018) 57:689–701. doi: 10.1007/s00394-016-1356-0 28070638 PMC5845591

[50] Westhoff E Wu X Kiemeney LA Lerner SP Ye Y Huang M. Dietary patterns and risk of recurrence and progression in non-muscle-invasive bladder cancer. Int J Cancer (2018) 142:1797–804. doi: 10.1002/ijc.31214 29235103

[51] Aveta A Cacciapuoti C Barone B Di Zazzo E Del Giudice F Maggi M. The impact of meat intake on bladder cancer incidence: is it really a relevant risk? Cancers (2022) 14:4775. doi: 10.3390/cancers14194775 36230700 PMC9564157

[52] Yu J Li H Liu Z Wang T Zhou F Ma S. Meat intake and the risk of bladder cancer: a systematic review and meta-analysis of observational studies. Nutr Cancer (2023) 75:825–45. doi: 10.1080/01635581.2022.2159043 36537666

[53] Dianatinasab M Wesselius A Salehi-Abargouei A Yu EYW Fararouei M Brinkman M. Dietary fats and their sources in association with the risk of bladder cancer: a pooled analysis of 11 prospective cohort studies. Int J Cancer (2022) 151:44–55. doi: 10.1002/ijc.33970 35182086 PMC9303525

[54] Leeming RC Karagas MR Gilbert-Diamond D Emond JA Zens MS Schned AR. Diet quality and survival in a population-based bladder cancer study. Nutr Cancer (2022) 74:2400–11. doi: 10.1080/01635581.2021.2008989 PMC9387520 34882045

[55] Catto JWF Rogers Z Downing A Mason SJ Jubber I Bottomley S. Lifestyle factors in patients with bladder cancer: a contemporary picture of tobacco smoking, electronic cigarette use, body mass index, and levels of physical activity. Eur Urol Focus (2023). doi: 10.1016/j.euf.2023.04.003 37080801

[56] Zhang W Yang F Mao S Wang R Chen H Ran Y. Bladder cancer-associated microbiota: recent advances and future perspectives. Heliyon (2023) 9:e13012. doi: 10.1016/j.heliyon.2023.e13012 36704283 PMC9871226

[57] Min K Kim HT Lee EH Park H Ha Y-S. Bacteria for treatment: microbiome in bladder cancer. Biomedicines (2022) 10:1783. doi: 10.3390/biomedicines10081783 35892683 PMC9332069

[58] Tan WS Teo CH Chan D Ang KM Heinrich M Feber A. Exploring patients’ experience and perception of being diagnosed with bladder cancer: a mixed-methods approach. BJU Int (2020) 125:669–78. doi: 10.1111/bju.15008 PMC7318301 31975539

[59] Edmondson AJ Birtwistle JC Catto JWF Twiddy M. The patients’ experience of a bladder cancer diagnosis: a systematic review of the qualitative evidence. J Cancer Surviv (2017) 11:453–61. doi: 10.1007/s11764-017-0603-6 PMC5500680 28213769

[60] Yu EY-W Nekeman D Billingham LJ James ND Cheng K Bryan RT. Health-related quality of life around the time of diagnosis in patients with bladder cancer. BJU Int (2019) 124:984–91. doi: 10.1111/bju.14804 PMC6907410 31077532

[61] Vaioulis A Bonotis K Perivoliotis K Kiouvrekis Y Gravas S Tzortzis V. Quality of life and anxiety in patients with first diagnosed non-muscle invasive bladder cancer who receive adjuvant bladder therapy. Bladder Cancer (2021) 7:297–306. doi: 10.3233/BLC-201524 PMC11181705 38993613

[62] González-Padilla DA González-Díaz A Guerrero-Ramos F Rodríguez-Serrano A García-Jarabo E Corona-laPuerta M. Quality of life and adverse events in patients with nonmuscle invasive bladder cancer receiving adjuvant treatment with BCG, MMC, or chemohyperthermia. Urol Oncol (2021) 39:76.e9–76.e14. doi: 10.1016/j.urolonc.2020.07.003 32753359

[63] Gontero P Oderda M Mehnert A Gurioli A Marson F Lucca I. The impact of intravesical gemcitabine and 1/3 dose bacillus calmette-guérin instillation therapy on the quality of life in patients with nonmuscle invasive bladder cancer: results of a prospective, randomized, phase II trial. J Urol (2013) 190:857–62. doi: 10.1016/j.juro.2013.03.097 23545101

[64] Beitz JM Zuzelo PR. The lived experience of having a neobladder. West J Nurs Res (2003) 25:294–316. doi: 10.1177/0193945902250417 12705113

[65] Nayak A Cresswell J Mariappan P. Quality of life in patients undergoing surveillance for non-muscle invasive bladder cancer-a systematic review. Transl Androl Urol (2021) 10:2737–49. doi: 10.21037/tau-20-1333 PMC8261437 34295759

[66] Mason SJ Downing A Wright P Hounsome L Bottomley SE Corner J. Health-related quality of life after treatment for bladder cancer in England. Br J Cancer (2018) 118:1518–28. doi: 10.1038/s41416-018-0084-z PMC5988662 29755116

[67] Catto JWF Downing A Mason S Wright P Absolom K Bottomley S. Quality of life after bladder cancer: a cross-sectional survey of patient-reported outcomes. Eur Urol (2021) 79:621–32. doi: 10.1016/j.eururo.2021.01.032 PMC8082273 33581875

[68] Tsai Y-S Wu T-Y Jou Y-C Tzai T-S Wang J-D. Determinants and dynamic changes of generic quality of life in human bladder cancer patients. J Clin Med (2021) 10:5472. doi: 10.3390/jcm10235472 34884174 PMC8658139

[69] Cooper OAE Ghatnekar O Piglowska N Smith CA Swinburn P Catto JWF. Elicitation of health state utilities associated with progression from bacillus calmette-guerin (BCG) unresponsive non-muscle invasive bladder cancer (NMIBC). PharmacoEconomics - Open (2023) 7:469–77. doi: 10.1007/s41669-023-00392-4 PMC9897878 36737511

[70] Clements MB Atkinson TM Dalbagni GM Li Y Vickers AJ Herr HW. Health-related quality of life for patients undergoing radical cystectomy: results of a large prospective cohort. Eur Urol (2022) 81:294–304. doi: 10.1016/j.eururo.2021.09.018 34629182 PMC8891075

[71] Yang LS Shan BL Shan LL Chin P Murray S Ahmadi N. A systematic review and meta-analysis of quality of life outcomes after radical cystectomy for bladder cancer. Surg Oncol (2016) 25:281–97. doi: 10.1016/j.suronc.2016.05.027 27566035

[72] Cox E Saramago P Kelly J Porta N Hall E Tan WS. Effects of bladder cancer on UK healthcare costs and patient health-related quality of life: evidence from the BOXIT trial. Clin Genitourin Cancer (2020) 18:e418–42. doi: 10.1016/j.clgc.2019.12.004 PMC7427321 32144049

[73] Smith AB McCabe S Deal AM Guo A Gessner KH Lipman R. Quality of life and health state utilities in bladder cancer. Bladder Cancer (2022) 8:55–70. doi: 10.3233/blc-211615 PMC11181821 38994519

[74] Botteman MF Pashos CL Redaelli A Laskin B Hauser R. The health economics of bladder cancer: a comprehensive review of the published literature. PharmacoEconomics (2003) 21:1315–30. doi: 10.1007/BF03262330 14750899

[75] Svatek RS Hollenbeck BK Holmäng S Lee R Kim SP Stenzl A. The economics of bladder cancer: costs and considerations of caring for this disease. Eur Urol (2014) 66:253–62. doi: 10.1016/j.eururo.2014.01.006 24472711

[76] Lee LJ Kwon CS Forsythe A Mamolo CM Masters ET Jacobs IA. Humanistic and economic burden of non-muscle invasive bladder cancer: results of two systematic literature reviews. Clin Outcomes Res (2020) 12:693–709. doi: 10.2147/CEOR.S274951 PMC7695604 33262624

[77] Mossanen M Wang Y Szymaniak J Tan WS Huynh MJ Preston MA. Evaluating the cost of surveillance for non-muscle-invasive bladder cancer: an analysis based on risk categories. World J Urol (2019) 37:2059–65. doi: 10.1007/s00345-018-2550-x 30446799

[78] Fujita N Hatakeyama S Momota M Narita T Tobisawa Y Yoneyama T. Single immediate instillation of chemotherapy is associated with decreased recurrence and progression in patients with high-risk non-muscle-invasive bladder cancer who receive adjuvant induction bacillus calmette-guérin therapy. Int J Urol (2022) 29:867–75. doi: 10.1111/iju.14926 35577361

[79] Gerace C Montorsi F Tambaro R Cartenì G De Luca S Tucci M. Cost of illness of urothelial bladder cancer in Italy. Clin Outcomes Res (2017) 9:433–42. doi: 10.2147/CEOR.S135065 PMC5533568 28769578

[80] Michaeli JC Boch T Albers S Michaeli T Michaeli DT. Socio-economic burden of disease: survivorship costs for bladder cancer. J Cancer Policy (2022) 32:100326. doi: 10.1016/j.jcpo.2022.100326 35560269

[81] Ortega-Ortega M Hanly P Pearce A Soerjomataram I Sharp L. Paid and unpaid productivity losses due to premature mortality from cancer in Europe in 2018. Int J Cancer (2022) 150:580–93. doi: 10.1002/ijc.33826 34569617

[82] Maisch P Retz M Gschwend JE Koll F Schmid SC. Clinical practice guidelines for bladder cancer: a systematic review and meta-analysis using the AGREE II instrument. Urol Int (2021) 105:31–40. doi: 10.1159/000509431 32829338

[83] Oswald D Pallauf M Herrmann TRW Netsch C Becker B Lehrich K. Transurethral resection of bladder tumors (TURBT). Urol Ausg A (2022) 61:71–82. doi: 10.1007/s00120-021-01741-z PMC8763753 34982181

[84] Teoh JY-C MacLennan S Chan VW-S Miki J Lee H-Y Chiong E. An international collaborative consensus statement on en bloc resection of bladder tumour incorporating two systematic reviews, a two-round Delphi survey, and a consensus meeting. Eur Urol (2020) 78:546–69. doi: 10.1016/j.eururo.2020.04.059 32389447

[85] Mariappan P Zachou A Grigor KM Edinburgh Uro-Oncology Group. Detrusor muscle in the first, apparently complete transurethral resection of bladder tumour specimen is a surrogate marker of resection quality, predicts risk of early recurrence, and is dependent on operator experience. Eur Urol (2010) 57:843–9. doi: 10.1016/j.eururo.2009.05.047 19524354

[86] Heer R Lewis R Duncan A Penegar S Vadiveloo T Clark E. Photodynamic versus white-light-guided resection of first-diagnosis non-muscle-invasive bladder cancer: PHOTO RCT. Health Technol Assess (2022) 26:1–144. doi: 10.3310/PLPU1526 PMC9639219 36300825

[87] Mowatt G Zhu S Kilonzo MM Boachie C Fraser C Griffiths TRL. Systematic review of the clinical effectiveness and cost-effectiveness of photodynamic diagnosis and urine biomarkers (FISH, ImmunoCyt, NMP22) and cytology for the detection and follow-up of bladder cancer. Health Technol Assess (2010) 14:1–356. doi: 10.3310/hta14040 20082749

[88] Daneshmand S Bazargani ST Bivalacqua TJ Holzbeierlein JM Willard B Taylor JM. Blue light cystoscopy for the diagnosis of bladder cancer: results from the US prospective multicenter registry. Urol Oncol (2018) 36:361.e1–6. doi: 10.1016/j.urolonc.2018.04.013 29859728

[89] Maisch P Koziarz A Vajgrt J Narayan V Kim MH Dahm P. Blue versus white light for transurethral resection of non-muscle invasive bladder cancer. Cochrane Database Syst Rev (2021) 12:CD013776. doi: 10.1002/14651858.CD013776.pub2 34850382 PMC8632646

[90] Chou R Selph S Buckley DI Fu R Griffin JC Grusing S. Comparative effectiveness of fluorescent versus white light cystoscopy for initial diagnosis or surveillance of bladder cancer on clinical outcomes: systematic review and meta-analysis. J Urol (2017) 197:548–58. doi: 10.1016/j.juro.2016.10.061 27780784

[91] Gravestock P Vale L Heer R PHOTO Trial Team. Reply to fredrik liedberg and Johannes bobjer’s letter to the Editor re: rakesh heer, Rebecca Lewis, thenmalar vadiveloo, et al. a randomized trial of PHOTOdynamic surgery in non-muscle-invasive bladder cancer. NEJM evid. in press. https://doi.org/10.1056/EVIDoa2200092. Eur Urol Open Sci (2022) 46:149–50. doi: 10.1016/j.euros.2022.10.019 PMC9676134 36420115

[92] Stenzl A Rouprêt M Witjes JA Gontero P. High-quality transurethral resection of bladder tumour needs additional forms of tumour delineation. Eur Urol (2023) 83:193–4. doi: 10.1016/j.eururo.2022.11.017 36473782

[93] Liedberg F Bobjer J. Re: rakesh heer, Rebecca Lewis, thenmalar vadiveloo, et al. a randomized trial of PHOTOdynamic surgery in non-muscle-invasive bladder cancer. NEJM evid. in press. https://doi.org/10.1056/EVIDoa2200092. Eur Urol Open Sci (2022) 46:53–4. doi: 10.1016/j.euros.2022.09.021 PMC9618787 36325368

[94] Iida K Naiki T Kawai N Etani T Ando R Ikegami Y. Bacillus calmette-guerin therapy after the second transurethral resection significantly decreases recurrence in patients with new onset high-grade T1 bladder cancer. BMC Urol (2016) 16:8. doi: 10.1186/s12894-016-0126-x 26920373 PMC4769574

[95] Kikuchi H Abe T Matsumoto R Osawa T Maruyama S Murai S. Outcomes of bacillus calmette–guérin therapy without a maintenance schedule for high-risk non-muscle-invasive bladder cancer in the second transurethral resection era. Int J Urol (2022) 29:251–8. doi: 10.1111/iju.14761 PMC9299795 34894009

[96] Regnier S Califano G Elalouf V Albisinni S Aziz A Di Trapani E. Restaging transurethral resection in Ta high-grade nonmuscle invasive bladder cancer: a systematic review. Curr Opin Urol (2022) 32:54–60. doi: 10.1097/MOU.0000000000000949 34812200

[97] Wettstein MS Baxter NN Sutradhar R Mamdani M Song P Qadri SR. Oncological benefit of re-resection for T1 bladder cancer: a comparative effectiveness study. BJU Int (2022) 129:258–68. doi: 10.1111/bju.15622 34674366

[98] Krajewski W Moschini M Nowak Ł Poletajew S Tukiendorf A Afferi L. Restaging transurethral resection of bladder tumours after BCG immunotherapy induction in patients with T1 non-muscle-invasive bladder cancer might not be associated with oncologic benefit. J Clin Med (2020) 9:E3306. doi: 10.3390/jcm9103306 PMC7602446 33076249

[99] Flaig TW Spiess PE Abern M Agarwal N Bangs R Boorjian SA. NCCN clinical practice guidelines in oncology (NCCN guidelines®): bladder cancer. Plymouth Meeting, PA: National Comprehensive Cancer Network (2022).

[100] Bosschieter J Nieuwenhuijzen JA Vis AN van Ginkel T Lissenberg-Witte BI Beckers GMA. An immediate, single intravesical instillation of mitomycin c is of benefit in patients with non-muscle-invasive bladder cancer irrespective of prognostic risk groups. Urol Oncol (2018) 36:400.e7–400.e14. doi: 10.1016/j.urolonc.2018.05.026 30064935

[101] Messing EM Tangen CM Lerner SP Sahasrabudhe DM Koppie TM Wood DP. Effect of intravesical instillation of gemcitabine vs saline immediately following resection of suspected low-grade non-muscle-invasive bladder cancer on tumor recurrence: SWOG S0337 randomized clinical trial. JAMA (2018) 319:1880–8. doi: 10.1001/jama.2018.4657 PMC6583489 29801011

[102] Sylvester RJ Oosterlinck W Holmang S Sydes MR Birtle A Gudjonsson S. Systematic review and individual patient data meta-analysis of randomized trials comparing a single immediate instillation of chemotherapy after transurethral resection with transurethral resection alone in patients with stage pTa-pT1 urothelial carcinoma of the bladder: which patients benefit from the instillation? Eur Urol (2016) 69:231–44. doi: 10.1016/j.eururo.2015.05.050 26091833

[103] Han RF Pan JG. Can intravesical bacillus calmette-guérin reduce recurrence in patients with superficial bladder cancer? a meta-analysis of randomized trials. Urology (2006) 67:1216–23. doi: 10.1016/j.urology.2005.12.014 16765182

[104] Lamm DL Blumenstein BA Crissman JD Montie JE Gottesman JE Lowe BA. Maintenance bacillus calmette-guerin immunotherapy for recurrent TA, T1 and carcinoma *in situ* transitional cell carcinoma of the bladder: a randomized southwest oncology group study. J Urol (2000) 163:1124–9. doi: 10.1016/S0022-5347(05)67707-5 10737480

[105] Lamm DL van der Meijden PM Morales A Brosman SA Catalona WJ Herr HW. Incidence and treatment of complications of bacillus calmette-guerin intravesical therapy in superficial bladder cancer. J Urol (1992) 147:596–600. doi: 10.1016/s0022-5347(17)37316-0 1538436

[106] Böhle A Jocham D Bock PR. Intravesical bacillus calmette-guerin versus mitomycin c for superficial bladder cancer: a formal meta-analysis of comparative studies on recurrence and toxicity. J Urol (2003) 169:90–5. doi: 10.1097/01.ju.0000039680.90768.b3 12478111

[107] Böhle A Bock PR. Intravesical bacille calmette-guérin versus mitomycin c in superficial bladder cancer: formal meta-analysis of comparative studies on tumor progression. Urology (2004) 63:682–686; discussion 686-687. doi: 10.1016/j.urology.2003.11.049 15072879

[108] Sylvester RJ van der Meijden APM Lamm DL. Intravesical bacillus calmette-guerin reduces the risk of progression in patients with superficial bladder cancer: a meta-analysis of the published results of randomized clinical trials. J Urol (2002) 168:1964–70. doi: 10.1097/01.ju.0000034450.80198.1c 12394686

[109] Malmström P-U Sylvester RJ Crawford DE Friedrich M Krege S Rintala E. An individual patient data meta-analysis of the long-term outcome of randomised studies comparing intravesical mitomycin c versus bacillus calmette-guérin for non–muscle-invasive bladder cancer. Eur Urol (2009) 56:247–56. doi: 10.1016/j.eururo.2009.04.038 19409692

[110] Tan GH Kuk C Zlotta AR. Are there differences among bacillus calmette-guérin (BCG) strains regarding their clinical efficacy in the treatment of non-muscleinvasive bladder cancer? the jury is still out but the answer is likely no. Can Urol Assoc J J Assoc Urol Can (2020) 14:E54–6. doi: 10.5489/cuaj.5923 PMC7012286 31348742

[111] Boehm BE Cornell JE Wang H Mukherjee N Oppenheimer JS Svatek RS. Efficacy of bacillus calmette-guérin strains for treatment of nonmuscle invasive bladder cancer: a systematic review and network meta-analysis. J Urol (2017) 198:503–10. doi: 10.1016/j.juro.2017.01.086 PMC6464123 28286068

[112] Del Giudice F Asero V Bologna E Scornajenghi CM Carino D Dolci V. Efficacy of different bacillus of calmette-guérin (BCG) strains on recurrence rates among Intermediate/High-risk non-muscle invasive bladder cancers (NMIBCs): single-arm study systematic review, cumulative and network meta-analysis. Cancers (2023) 15:1937. doi: 10.3390/cancers15071937 37046598 PMC10093360

[113] Kamat AM Flaig TW Grossman HB Konety B Lamm D O’Donnell MA. Expert consensus document: consensus statement on best practice management regarding the use of intravesical immunotherapy with BCG for bladder cancer. Nat Rev Urol (2015) 12:225–35. doi: 10.1038/nrurol.2015.58 25800393

[114] Kamat AM Porten S. Myths and mysteries surrounding bacillus calmette-guérin therapy for bladder cancer. Eur Urol (2014) 65:267–9. doi: 10.1016/j.eururo.2013.10.016 PMC4676267 24199712

[115] Tabayoyong WB Kamat AM O’Donnell MA McKiernan JM Ray-Zack MD Palou J. Systematic review on the utilization of maintenance intravesical chemotherapy in the management of non-muscle-invasive bladder cancer. Eur Urol Focus (2018) 4:512–21. doi: 10.1016/j.euf.2018.08.019 PMC7810510 30190111

[116] Ehdaie B Sylvester R Herr HW. Maintenance bacillus calmette-guérin treatment of non–muscle-invasive bladder cancer: a critical evaluation of the evidence. Eur Urol (2013) 64:579–85. doi: 10.1016/j.eururo.2013.05.027 23711538

[117] Martínez-Piñeiro L Portillo JA Fernández JM Zabala JA Cadierno I Moyano JL. Maintenance therapy with 3-monthly bacillus calmette-guérin for 3 years is not superior to standard induction therapy in high-risk non-muscle-invasive urothelial bladder carcinoma: final results of randomised CUETO study 98013. Eur Urol (2015) 68:256–62. doi: 10.1016/j.eururo.2015.02.040 25794457

[118] Palou J Laguna P Millán-Rodríguez F Hall RR Salvador-Bayarri J Vicente-Rodríguez J. Control group and maintenance treatment with bacillus calmette-guerin for carcinoma *in situ* and/or high grade bladder tumors. J Urol (2001) 165:1488–91. doi: 10.1016/S0022-5347(05)66333-1 11342902

[119] Grimm M-O van der Heijden AG Colombel M Muilwijk T Martínez-Piñeiro L Babjuk MM. Treatment of high-grade non-muscle-invasive bladder carcinoma by standard number and dose of BCG instillations versus reduced number and standard dose of BCG instillations: results of the European association of urology research foundation randomised phase III clinical trial “NIMBUS”. Eur Urol (2020) 78:690–8. doi: 10.1016/j.eururo.2020.04.066 32446864

[120] Palou Redorta J Solsona E Angulo J Fernández JM Madero R Unda M. Retrospective study of various conservative treatment options with bacille calmette-guérin in bladder urothelial carcinoma T1G3: maintenance therapy. Actas Urol Esp (2016) 40:370–7. doi: 10.1016/j.acuro.2015.12.009 26922518

[121] Miyake M Iida K Nishimura N Miyamoto T Fujimoto K Tomida R. Non-maintenance intravesical bacillus calmette-guérin induction therapy with eight doses in patients with high- or highest-risk non-muscle invasive bladder cancer: a retrospective non-randomized comparative study. BMC Cancer (2021) 21:266. doi: 10.1186/s12885-021-07966-7 33706705 PMC7948348

[122] Zhu S Tang Y Li K Shang Z Jiang N Nian X. Optimal schedule of bacillus calmette-guerin for non-muscle-invasive bladder cancer: a meta-analysis of comparative studies. BMC Cancer (2013) 13:332. doi: 10.1186/1471-2407-13-332 23829273 PMC3722001

[123] Chen S Zhang N Shao J Wang X. Maintenance versus non-maintenance intravesical bacillus calmette-guerin instillation for non-muscle invasive bladder cancer: a systematic review and meta-analysis of randomized clinical trials. Int J Surg (2018) 52:248–57. doi: 10.1016/j.ijsu.2018.02.045 29499363

[124] Oddens J Brausi M Sylvester R Bono A van de Beek C van Andel G. Final results of an EORTC-GU cancers group randomized study of maintenance bacillus calmette-guérin in intermediate- and high-risk Ta, T1 papillary carcinoma of the urinary bladder: one-third dose versus full dose and 1 year versus 3 years of maintenance. Eur Urol (2013) 63:462–72. doi: 10.1016/j.eururo.2012.10.039 23141049

[125] Taylor J Becher E Steinberg GD. Update on the guideline of guidelines: non-muscle-invasive bladder cancer. BJU Int (2020) 125:197–205. doi: 10.1111/bju.14915 31597003

[126] Laukhtina E Abufaraj M Al-Ani A Ali MR Mori K Moschini M. Intravesical therapy in patients with intermediate-risk non-muscle-invasive bladder cancer: a systematic review and network meta-analysis of disease recurrence. Eur Urol Focus (2022) 8:447–56. doi: 10.1016/j.euf.2021.03.016 33762203

[127] Schmidt S Kunath F Coles B Draeger DL Krabbe L-M Dersch R. Intravesical bacillus calmette-guérin versus mitomycin c for Ta and T1 bladder cancer. Cochrane Database Syst Rev (2020) 1:CD011935. doi: 10.1002/14651858.CD011935.pub2 31912907 PMC6956215

[128] Lindgren MS Hansen E Azawi N Nielsen AM Dyrskjøt L Jensen JB. DaBlaCa-13 study: oncological outcome of short-term, intensive chemoresection with mitomycin in nonmuscle invasive bladder cancer: primary outcome of a randomized controlled trial. J Clin Oncol (2023) 41:206–11. doi: 10.1200/JCO.22.00470 36223555

[129] Matulewicz RS Steinberg GD. Non-muscle-invasive bladder cancer: overview and contemporary treatment landscape of neoadjuvant chemoablative therapies. Rev Urol (2020) 22:43–51. 32760227 PMC7393683

[130] McElree IM Steinberg RL Martin AC Richards J Mott SL Gellhaus PT. Sequential intravesical gemcitabine and docetaxel for bacillus calmette-guérin-naïve high-risk nonmuscle-invasive bladder cancer. J Urol (2022) 208:589–99. doi: 10.1097/JU.0000000000002740 35892270

[131] Chevuru PT McElree IM Mott SL Steinberg RL O’Donnell MA Packiam VT. Long-term follow-up of sequential intravesical gemcitabine and docetaxel salvage therapy for non-muscle invasive bladder cancer. Urol Oncol (2023) 41:148.e1–7. doi: 10.1016/j.urolonc.2022.10.030 36456454

[132] McElree IM Steinberg RL Mott SL O’Donnell MA Packiam VT. Comparison of sequential intravesical gemcitabine and docetaxel vs bacillus calmette-guérin for the treatment of patients with high-risk non-muscle-invasive bladder cancer. JAMA Netw Open (2023) 6:e230849. doi: 10.1001/jamanetworkopen.2023.0849 36853609 PMC9975907

[133] Kawada T Yanagisawa T Araki M Pradere B Shariat SF. Sequential intravesical gemcitabine and docetaxel therapy in patients with nonmuscle invasive bladder cancer: a systematic review and meta-analysis. Curr Opin Urol (2023) 33:211–8. doi: 10.1097/MOU.0000000000001065 36482766

[134] Zhao H Chan VW-S Castellani D Chan EO-T Ong WLK Peng Q. Intravesical chemohyperthermia vs. bacillus calmette-guerin instillation for intermediate- and high-risk non-muscle invasive bladder cancer: a systematic review and meta-analysis. Front Surg (2021) 8:775527. doi: 10.3389/fsurg.2021.775527 34888347 PMC8649716

[135] Guerrero-Ramos F González-Padilla DA González-Díaz A de la Rosa-Kehrmann F Rodríguez-Antolín A Inman BA. Recirculating hyperthermic intravesical chemotherapy with mitomycin c (HIVEC) versus BCG in high-risk non-muscle-invasive bladder cancer: results of the HIVEC-HR randomized clinical trial. World J Urol (2022) 40:999–1004. doi: 10.1007/s00345-022-03928-1 35037963 PMC8994727

[136] Plata A Guerrero-Ramos F Garcia C González-Díaz A Gonzalez-Valcárcel I de la Morena JM. Long-term experience with hyperthermic chemotherapy (HIVEC) using mitomycin-c in patients with non-muscle invasive bladder cancer in Spain. J Clin Med (2021) 10:5105. doi: 10.3390/jcm10215105 34768625 PMC8584886

[137] Tan WS Steinberg G Witjes JA Li R Shariat SF Roupret M. Intermediate-risk non-muscle-invasive bladder cancer: updated consensus definition and management recommendations from the international bladder cancer group. Eur Urol Oncol (2022) 5:505–16. doi: 10.1016/j.euo.2022.05.005 35718695

[138] de Jong JJ Hendricksen K Rosier M Mostafid H Boormans JL. Hyperthermic intravesical chemotherapy for BCG unresponsive non-muscle invasive bladder cancer patients. Bladder Cancer (2018) 4:395–401. doi: 10.3233/BLC-180191 30417050 PMC6218110

[139] Pijpers OM Hendricksen K Mostafid H de Jong FC Rosier M Mayor N. Long-term efficacy of hyperthermic intravesical chemotherapy for BCG-unresponsive non-muscle invasive bladder cancer. Urol Oncol (2022) 40:62.e13–20. doi: 10.1016/j.urolonc.2021.07.019 34470725

[140] Tan WS Kelly JD. Intravesical device-assisted therapies for non-muscle-invasive bladder cancer. Nat Rev Urol (2018) 15:667–85. doi: 10.1038/s41585-018-0092-z 30254383

[141] Sousa A Piñeiro I Rodríguez S Aparici V Monserrat V Neira P. Recirculant hyperthermic IntraVEsical chemotherapy (HIVEC) in intermediate-high-risk non-muscle-invasive bladder cancer. Int J Hyperthermia (2016) 32:374–80. doi: 10.3109/02656736.2016.1142618 26915466

[142] Melgarejo-Segura MT Morales-Martínez A Yáñez-Castillo Y Arrabal-Polo MÁ Gómez-Lechuga P Pareja-Vílchez M. A systematic review of the efficacy of intravesical electromotive drug administration therapy for non-muscle invasive bladder cancer. Urol Oncol (2023) 41:166–76. doi: 10.1016/j.urolonc.2022.09.016 36328923

[143] Zhou W Liu J Mao D Hu C Gao D. The clinical efficacy and safety of equipment-assisted intravesical instillation of mitomycin c after transurethral resection of bladder tumour in patients with nonmuscular invasive bladder cancer: a meta-analysis. PloS One (2022) 17:e0276453. doi: 10.1371/journal.pone.0276453 36269742 PMC9586381

[144] Arrabal Polo MÁ Melgarejo Segura MT Yáñez Castillo Y Morales Martínez A Pareja Vílchez M Arrabal Martín M. Adjuvant intravesical treatment in patients with intermediate and high-risk non-muscle-invasive bladder cancer with BCG versus MMC applied with COMBAT or EMDA. results of a prospective study. J Cancer Res Clin Oncol (2023), 1–7. doi: 10.1007/s00432-023-04688-0 PMC10035471 36952006

[145] Melgarejo Segura MT Morales Martínez A Yáñez Castillo Y Arrabal Polo MÁ Gómez Lechuga P Pareja Vílchez M. Conductive hyperthermic chemotherapy versus electromotive drug administration of mitomycin c as intravesical adjuvant treatment of patients with intermediate or high-risk non-muscle invasive bladder cancer. Urol Oncol (2023) 41:109.e1–8. doi: 10.1016/j.urolonc.2022.10.019 36379812

[146] Jung JH Gudeloglu A Kiziloz H Kuntz GM Miller A Konety BR. Intravesical electromotive drug administration for non-muscle invasive bladder cancer. Cochrane Database Syst Rev (2017) 9:CD011864. doi: 10.1002/14651858.CD011864.pub2 28898400 PMC6483767

[147] Gontero P Dominguez-Escrig JL Rhijn BW Mostafid AH Rouprêt M Cohen D. Assessing the impact of BCG on progression of NMIBC in the new EAU high risk and very high-risk groups. Eur Urol (2022) 81:S366–7. doi: 10.1016/S0302-2838(22)00321-9

[148] Catto JWF Gordon K Collinson M Poad H Twiddy M Johnson M. Radical cystectomy against intravesical BCG for high-risk high-grade nonmuscle invasive bladder cancer: results from the randomized controlled BRAVO-feasibility study. J Clin Oncol (2021) 39:202–14. doi: 10.1200/JCO.20.01665 PMC8078404 33332191

[149] Deininger S Törzsök P Mitterberger M Pallauf M Oswald D Deininger C. From interferon to checkpoint inhibition therapy-a systematic review of new immune-modulating agents in bacillus calmette-guérin (BCG) refractory non-muscle-invasive bladder cancer (NMIBC). Cancers (2022) 14:694. doi: 10.3390/cancers14030694 35158964 PMC8833656

[150] García-Perdomo HA Sánchez AL Spiess PE. Immune checkpoints inhibitors in the management of high-risk non-muscle-invasive bladder cancer. a scoping review. Urol Oncol (2022) 40:409.e1–8. doi: 10.1016/j.urolonc.2022.02.003 35232680

[151] Al Hussein Al Awamlh B Chang SS. Novel therapies for high-risk non-muscle invasive bladder cancer. Curr Oncol Rep (2023) 25:83–91. doi: 10.1007/s11912-022-01350-9 36571706 PMC9791638

[152] Mariappan P Johnston A Padovani L Clark E Trail M Hamid S. Enhanced quality and effectiveness of transurethral resection of bladder tumour in non-muscle-invasive bladder cancer: a multicentre real-world experience from scotland’s quality performance indicators programme. Eur Urol (2020) 78:520–30. doi: 10.1016/j.eururo.2020.06.051 32690321

[153] Miyake M Kikuchi E Shinozaki K Piao Y Hayashi N Koto R. Real-world treatment patterns and clinical outcomes of Japanese patients with non-muscle invasive bladder cancer receiving intravesical bacillus calmette-guérin treatment. Int J Urol (2022) 29:1120–9. doi: 10.1111/iju.14933 PMC9790662 35598101

[154] Mariappan P Johnston A Trail M Hamid S Hollins G Dreyer BA. Multicentre real world long-term outcomes in 2773 primary non-muscle-invasive bladder cancer (NMIBC) patients managed within the Scottish bladder cancer quality performance indicator programme. Eur Urol (2022) 81:S242–3. doi: 10.1016/S0302-2838(22)00243-3

[155] Krajewski W Moschini M Chorbińska J Nowak Ł Poletajew S Tukiendorf A. Delaying BCG immunotherapy onset after transurethral resection of non-muscle-invasive bladder cancer is associated with adverse survival outcomes. World J Urol (2021) 39:2545–52. doi: 10.1007/s00345-020-03522-3 PMC8332577 33230571

[156] Mori K Miura N Babjuk M Karakiewicz PI Mostafaei H Laukhtina E. Low compliance to guidelines in nonmuscle-invasive bladder carcinoma: a systematic review. Urol Oncol (2020) 38:774–82. doi: 10.1016/j.urolonc.2020.06.013 32654948

[157] Matulay JT Tabayoyong W Duplisea JJ Chang C Daneshmand S Gore JL. Variability in adherence to guidelines based management of nonmuscle invasive bladder cancer among society of urologic oncology (SUO) members. Urol Oncol (2020) 38:796.e1–6. doi: 10.1016/j.urolonc.2020.04.026 32430255

[158] Balakrishnan AS Washington SL Meng MV Porten SP. Determinants of guideline-based treatment in patients with cT1 bladder cancer. Clin Genitourin Cancer (2019) 17:e461–71. doi: 10.1016/j.clgc.2019.01.007 30799130

[159] Dunsmore J Duncan E Mariappan P de Bruin M MacLennan S Dimitropoulos K. What influences adherence to guidance for postoperative instillation of intravesical chemotherapy to patients with bladder cancer? BJU Int (2021) 128:225–35. doi: 10.1111/bju.15336 33450116

[160] Choo SH Nishiyama H Kitamura H Chen C-H Pu Y-S Lee H-L. Practice pattern of non-muscle invasive bladder cancer in Japan, Korea and Taiwan: a web-based survey. Int J Urol (2019) 26:1121–7. doi: 10.1111/iju.14105 31512280

[161] Jeglinschi S Schirmann A Durand M Sanchez S Larré S Léon P. Factors affecting guideline adherence in the initial treatment of non-muscle invasive bladder cancer: retrospective study in a French peripheral hospital. Prog En Urol (2020) 30:26–34. doi: 10.1016/j.purol.2019.11.003 31813714

[162] Wang D-Q Huang Q Huang X Jin Y-H Wang Y-Y Shi Y-X. Knowledge of and compliance with guidelines in the management of non-muscle-invasive bladder cancer: a survey of Chinese urologists. Front Oncol (2021) 11:735704. doi: 10.3389/fonc.2021.735704 34778048 PMC8580413

[163] Downs TM Weissert JA Ravvaz K. Can we improve nonmuscle invasive bladder cancer guideline adherence with smarter risk stratification? J Urol (2018) 200:706–8. doi: 10.1016/j.juro.2018.06.019 29906436

[164] Ferro M Tătaru OS Musi G Lucarelli G Abu Farhan AR Cantiello F. Modified Glasgow prognostic score as a predictor of recurrence in patients with high grade non-muscle invasive bladder cancer undergoing intravesical bacillus calmette-guerin immunotherapy. Diagnostics (2022) 12:586. doi: 10.3390/diagnostics12030586 35328139 PMC8947693

[165] Plasek J Weissert J Downs T Richards K Ravvaz K. Clinicopathological criteria predictive of recurrence following bacillus calmette-guérin therapy initiation in non-muscle-invasive bladder cancer: retrospective cohort study. JMIR Cancer (2021) 7:e25800. doi: 10.2196/25800 34156341 PMC8277393

[166] Mariappan P. Project collaborators. the Scottish bladder cancer quality performance indicators influencing outcomes, prognosis, and surveillance (Scot BC quality OPS) clinical project. Eur Urol Focus (2021) 7:905–8. doi: 10.1016/j.euf.2021.07.011 34419380

[167] Beardo P Pinto R Ayerra H Agüera J Armijos S Álvarez-Ossorio JL. Optimizing treatment for non muscle-invasive bladder cancer with an app. Actas Urol Esp (2022) 46:230–7. doi: 10.1016/j.acuroe.2021.12.010 35307306

[168] Beeren I de Goeij L Dandis R Vidra N van Zutphen M Witjes JA. Limited changes in lifestyle behaviours after non-muscle invasive bladder cancer diagnosis. Cancers (2022) 14:960. doi: 10.3390/cancers14040960 35205711 PMC8869990

[169] van Osch FHM Jochems SHJ Reulen RC Pirrie SJ Nekeman D Wesselius A. The association between smoking cessation before and after diagnosis and non-muscle-invasive bladder cancer recurrence: a prospective cohort study. Cancer Causes Control (2018) 29:675–83. doi: 10.1007/s10552-018-1046-8 PMC5999150 29846846

[170] Yuruk E Tuken M Colakerol A Serefoglu EC. The awareness of patients with non-muscle invasive bladder cancer regarding the importance of smoking cessation and their access to smoking cessation programs. Int Braz J Urol (2017) 43:607–14. doi: 10.1590/S1677-5538.IBJU.2016.0014 PMC5557435 28537702

[171] Westhoff E Kampman E Aben KK Hendriks IG Witjes JA Kiemeney LA. Low awareness, adherence, and practice but positive attitudes regarding lifestyle recommendations among non-muscle-invasive bladder cancer patients. Urol Oncol (2019) 37:573.e1–8. doi: 10.1016/j.urolonc.2019.04.016 31076356

[172] van Zutphen M Hof JP Aben KKH Kampman E Witjes JA Kiemeney LA. Adherence to lifestyle recommendations after non-muscle invasive bladder cancer diagnosis and risk of recurrence. Am J Clin Nutr (2023) 117:681–90. doi: 10.1016/j.ajcnut.2022.12.022 36781128

[173] Vidra N Beeren I van Zutphen M Aben KK Kampman E Witjes JA. Longitudinal associations of adherence to lifestyle recommendations and health-related quality of life in patients with non-muscle invasive bladder cancer. Int J Cancer (2023) 152:2032–42. doi: 10.1002/ijc.34418 36594579

[174] Abushamma F Khayyat Z Soroghle A Zyoud SH Jaradat A Akkawi M. The impact of non-compliance to a standardized risk-adjusted protocol on recurrence, progression, and mortality in non-muscle invasive bladder cancer. Cancer Manag Res (2021) 13:2937–45. doi: 10.2147/CMAR.S299148 PMC8020126 33833577

[175] Alhogbani MM Picard JA Fassi-Fehri MH Badet JL Colombel CM. Prognostic impact of bacillus calmette-guérin interruption at the time of induction and consolidation. Urol Ann (2017) 9:315–20. doi: 10.4103/UA.UA_115_17 PMC5656953 29118530

[176] Nummi A Järvinen R Sairanen J Huotari K. A retrospective study on tolerability and complications of bacillus calmette-guérin (BCG) instillations for non-muscle-invasive bladder cancer. Scand J Urol (2019) 53:116–22. doi: 10.1080/21681805.2019.1609080 31074322

[177] Tapiero S Helfand A Kedar D Yossepowitch O Nadu A Baniel J. Patient compliance with maintenance intravesical therapy for nonmuscle invasive bladder cancer. Urology (2018) 118:107–13. doi: 10.1016/j.urology.2018.04.039 29792974

[178] Serretta V Scalici Gesolfo C Alonge V Cicero G Moschini M Colombo R. Does the compliance to intravesical BCG differ between common clinical practice and international multicentric trials? Urol Int (2016) 96:20–4. doi: 10.1159/000430501 26201964

[179] Datovo JCF Neto WA Mendonça GB Andrade DL Reis LO. Prognostic impact of non-adherence to follow-up cystoscopy in non-muscle-invasive bladder cancer (NMIBC). World J Urol (2019) 37:2067–71. doi: 10.1007/s00345-019-02697-8 30805685

[180] van der Meijden APM Sylvester RJ Oosterlinck W Hoeltl W Bono AV. Maintenance bacillus calmette-guerin for Ta T1 bladder tumors is not associated with increased toxicity: results from a European organisation for research and treatment of cancer genito-urinary group phase III trial. Eur Urol (2003) 44:429–34. doi: 10.1016/S0302-2838(03)00357-9 14499676

[181] Brausi M Oddens J Sylvester R Bono A van de Beek C van Andel G. Side effects of bacillus calmette-guérin (BCG) in the treatment of intermediate- and high-risk Ta, T1 papillary carcinoma of the bladder: results of the EORTC genito-urinary cancers group randomised phase 3 study comparing one-third dose with full dose and 1 year with 3 years of maintenance BCG. Eur Urol (2014) 65:69–76. doi: 10.1016/j.eururo.2013.07.021 23910233

[182] Nurminen P Ettala O Uusitalo-Seppälä R Nummi A Järvinen R Antti K. Incidence of and mortality from bacille calmette-guérin (BCG) infections after BCG instillation therapy. BJU Int (2021) 129:737–43. doi: 10.1111/bju.15608 34617382

[183] Alshyarba MH Alamri A Assiri AA. Economic impacts of the bacillus calmette-guérin (BCG) therapy shortage and the proposed solutions for patients with non-muscle invasive bladder cancer in aseer province, Saudi Arabia. J Fam Med Prim Care (2020) 9:2758–62. doi: 10.4103/jfmpc.jfmpc_171_20 PMC7491787 32984121

[184] Pérez-Aizpurua X Monzó-Gardiner JI Maqueda-Arellano J Buendía-González E Cuello-Sánchez L Tufet I Jaumot JJ. BCG Shortage for intravesical instillation is associated with early tumoral recurrence in patients with high-risk non-muscle invasive bladder tumours. Actas Urol Esp (2023) 47:250–8. doi: 10.1016/j.acuroe.2023.01.005 36754206

[185] Ourfali S Ohannessian R Fassi-Fehri H Pages A Badet L Colombel M. Recurrence rate and cost consequence of the shortage of bacillus calmette-guérin connaught strain for bladder cancer patients. Eur Urol Focus (2021) 7:111–6. doi: 10.1016/j.euf.2019.04.002 31005491

[186] Lee S Lim B You D Hong B Hong JH Kim C-S. Association of bacillus calmette-guerin shortages with bladder cancer recurrence: a single-center retrospective study. Urol Oncol (2020) 38:851.e11–851.e17. doi: 10.1016/j.urolonc.2020.07.014 32800440

[187] Ferro M Del Giudice F Carrieri G Busetto GM Cormio L Hurle R. The impact of SARS-CoV-2 pandemic on time to primary, secondary resection and adjuvant intravesical therapy in patients with high-risk non-muscle invasive bladder cancer: a retrospective multi-institutional cohort analysis. Cancers (2021) 13:5276. doi: 10.3390/cancers13215276 34771440 PMC8582553

[188] Culpan M Keser F Acar HC Otunctemur A Kucuk EV Erdem S. Impact of delay in cystoscopic surveillance on recurrence and progression rates in patients with non-muscle-invasive bladder cancer during the COVID-19 pandemic. Int J Clin Pract (2021) 75:e14490. doi: 10.1111/ijcp.14490 34117682 PMC8420249

[189] van Hoogstraten LMC Kiemeney LA Meijer RP van Leenders GJLH Vanneste BGL Incrocci L. The impact of the COVID-19 pandemic on bladder cancer care in the Netherlands. Bladder Cancer (2022) 8:139–54. doi: 10.3233/blc-211608 PMC11181832 38993368

[190] Decaestecker K Oosterlinck W. Managing the adverse events of intravesical bacillus calmette–guérin therapy. Res Rep Urol (2015) 7:157–63. doi: 10.2147/RRU.S63448 PMC4630183 26605208

[191] Rutherford C Patel MI Tait M-A Smith DP Costa DSJ Sengupta S. Patient-reported outcomes in non-muscle invasive bladder cancer: a mixed-methods systematic review. Qual Life Res (2021) 30:345–66. doi: 10.1007/s11136-020-02637-9 32960394

[192] Balasubramanian A Gunjur A Weickhardt A Papa N Bolton D Lawrentschuk N. Adjuvant therapies for non-muscle-invasive bladder cancer: advances during BCG shortage. World J Urol (2022) 40:1111–24. doi: 10.1007/s00345-021-03908-x 35083522

[193] Moon YJ Cho KS Jeong JY Chung DY Kang DH Jung HD. Effects of intravesical BCG maintenance therapy duration on recurrence rate in high-risk non-muscle invasive bladder cancer (NMIBC): systematic review and network meta-analysis according to EAU COVID-19 recommendations. PloS One (2022) 17:e0273733. doi: 10.1371/journal.pone.0273733 36074771 PMC9455878

[194] Bree KK Shan Y Hensley PJ Lobo N Hu C Tyler DS. Management, surveillance patterns, and costs associated with low-grade papillary stage Ta non-muscle-invasive bladder cancer among older adults, 2004-2013. JAMA Netw Open (2022) 5:e223050. doi: 10.1001/jamanetworkopen.2022.3050 35302627 PMC8933744

[195] Paciotti M Contieri R Fasulo V Casale P Saita A Buffi NM. Active surveillance for recurrent non-muscle invasive bladder cancer: which lessons have we learned during COVID-19 pandemic? Minerva Urol Nefrol (2022) 74:1–4. doi: 10.23736/S2724-6051.21.04613-9 34338493

[196] Tayeh GA Khalil N Alkassis M Aoun F Waked C Nemr E. Urothelial carcinoma in COVID-19: lessons from a pandemic and their impact on clinical practice. Future Oncol (2021) 17:4233–5. doi: 10.2217/fon-2021-0895 PMC8547276 34672692

[197] Quan Y Jeong CW Kwak C Kim HH Kim HS Ku JH. Dose, duration and strain of bacillus calmette-guerin in the treatment of nonmuscle invasive bladder cancer: meta-analysis of randomized clinical trials. Med (Baltimore) (2017) 96:e8300. doi: 10.1097/MD.0000000000008300 PMC5662397 29049231

[198] Koch GE Smelser WW Chang SS. Side effects of intravesical BCG and chemotherapy for bladder cancer: what they are and how to manage them. Urology (2021) 149:11–20. doi: 10.1016/j.urology.2020.10.039 33181123

[199] Mathes J Todenhöfer T. Managing toxicity of intravesical therapy. Eur Urol Focus (2018) 4:464–7. doi: 10.1016/j.euf.2018.09.009 30473130

[200] Luckenbaugh AN Marks RM Miller DC Weizer AZ Stoffel JT Montgomery JS. A management algorithm for mitomycin c induced cystitis. Bladder Cancer Amst Neth (2017) 3:133–8. doi: 10.3233/BLC-160089 PMC5409048 28516158

[201] Shelley MD Mason MD Kynaston H. Intravesical therapy for superficial bladder cancer: a systematic review of randomised trials and meta-analyses. Cancer Treat Rev (2010) 36:195–205. doi: 10.1016/j.ctrv.2009.12.005 20079574

[202] Subiela JD Rodríguez Faba Ó Aumatell J Gonzalez-Padilla DA Rosales Bordes A Huguet J. Long-term recurrence and progression patterns in a contemporary series of patients with carcinoma *in situ* of the bladder with or without associated Ta/T1 disease treated with bacillus calmette-guérin: implications for risk-adapted follow-up. Eur Urol Focus (2023) 9:325–32. doi: 10.1016/j.euf.2022.09.007 36163105

[203] Ferro M Barone B Crocetto F Lucarelli G Busetto GM Del Giudice F. Predictive clinico-pathological factors to identify BCG, unresponsive patients, after re-resection for T1 high grade non-muscle invasive bladder cancer. Urol Oncol (2022) 40:490.e13–490.e20. doi: 10.1016/j.urolonc.2022.05.016 35676172

[204] Pijpers OM Van Hoogstraten LMC De Beijert I Oddens J Witjes JA Kiemeney LA. A0280 - nation-wide progression outcomes after BCG therapy for high- and very high-risk T1 non-muscle invasive bladder cancer patients – a prospective registry study. Eur Urol (2023) 83:S403–4. doi: 10.1016/S0302-2838(23)00329-9

[205] Lonati C Baumeister P Afferi L Mari A Minervini A Krajewski W. Survival outcomes after immediate radical cystectomy versus conservative management with bacillus calmette-guérin among T1 high-grade micropapillary bladder cancer patients: results from a multicentre collaboration. Eur Urol Focus (2022) 8:1270–7. doi: 10.1016/j.euf.2021.07.015 34419381

[206] Angulo JC Álvarez-Ossorio JL Domínguez-Escrig JL Moyano JL Sousa A Fernández JM. Hyperthermic mitomycin c in intermediate-risk non-muscle-invasive bladder cancer: results of the HIVEC-1 trial. Eur Urol Oncol (2023) 6:58–66. doi: 10.1016/j.euo.2022.10.008 36435738

[207] van Wijngaarden C Bus MTJ Ruiter AEC Lagerveld BW. A 6-month maintenance schedule of mitomycin c after radical nephroureterectomy for upper tract urothelial carcinoma for the prevention of intravesical recurrence: a retrospective, single center study. World J Urol (2023) 41:1077–83. doi: 10.1007/s00345-023-04316-z 36790518

[208] Gierth M Breyer J Zeman F Fritsche HM Cordes J Karl A. The HELENA study: hexvix®-TURB vs. white-light TURB followed by intravesical adjuvant chemotherapy-a prospective randomized controlled open-label multicenter non-inferiority study. World J Urol (2021) 39:3799–805. doi: 10.1007/s00345-021-03719-0 PMC8521513 34002265

[209] Nativ O Witjes JA Hendricksen K Cohen M Kedar D Sidi A. Combined thermo-chemotherapy for recurrent bladder cancer after bacillus calmette-guerin. J Urol (2009) 182:1313–7. doi: 10.1016/j.juro.2009.06.017 19683278

[210] Friedrich MG Pichlmeier U Schwaibold H Conrad S Huland H. Long-term intravesical adjuvant chemotherapy further reduces recurrence rate compared with short-term intravesical chemotherapy and short-term therapy with bacillus calmette-guérin (BCG) in patients with non-muscle-invasive bladder carcinoma. Eur Urol (2007) 52:1123–9. doi: 10.1016/j.eururo.2007.02.063 17383080

[211] Veskimae E Subbarayan S Campi R Carron D Omar MI Yuan C. A systematic review of outcome reporting, definition and measurement heterogeneity in non-muscle invasive bladder cancer effectiveness trials of adjuvant, prophylactic treatment after transurethral resection. Bladder Cancer (2021) 7:221–41. doi: 10.3233/BLC-201510 PMC11181687 38994538

[212] Fletcher SA Bivalacqua TJ Brawley OW Kates M. Race, ethnicity, and gender reporting in north American clinical trials for BCG-unresponsive non-muscle invasive bladder cancer. Urol Oncol (2022) 40:195.e13–195.e18. doi: 10.1016/j.urolonc.2021.11.015 34949513

[213] Javier-DesLoges J Nelson TJ Murphy JD McKay RR Stewart TF Kader AK. An evaluation of trends in the representation of patients by age, sex, and diverse race/ethnic groups in bladder and kidney cancer clinical trials. Urol Oncol (2022) 40:199.e15–199.e21. doi: 10.1016/j.urolonc.2022.03.013 PMC10441556 35431133

[214] Roumiguié M Kamat AM Bivalacqua TJ Lerner SP Kassouf W Böhle A. International bladder cancer group consensus statement on clinical trial design for patients with bacillus calmette-guérin-exposed high-risk non-muscle-invasive bladder cancer. Eur Urol (2022) 82:34–46. doi: 10.1016/j.eururo.2021.12.005 34955291

[215] Sharma A Palaniappan L. Improving diversity in medical research. Nat Rev Dis Primer (2021) 7:74. doi: 10.1038/s41572-021-00316-8 34650078

[216] Jobczyk M Stawiski K Kaszkowiak M Rajwa P Różański W Soria F. Deep learning-based recalibration of the CUETO and EORTC prediction tools for recurrence and progression of non-muscle-invasive bladder cancer. Eur Urol Oncol (2022) 5:109–12. doi: 10.1016/j.euo.2021.05.006 34092528

[217] Lucas M Jansen I van Leeuwen TG Oddens JR de Bruin DM Marquering HA. Deep learning-based recurrence prediction in patients with non-muscle-invasive bladder cancer. Eur Urol Focus (2022) 8:165–72. doi: 10.1016/j.euf.2020.12.008 33358370

[218] Muzaail HH El-Assmy A Harraz AM Awadalla A Shokeir AA Abdel-Aziz AF. Prediction of recurrence of non-muscle invasive bladder cancer: the role of androgen receptor and miRNA-2909. Urol Oncol (2022) 40:197.e25–197.e35. doi: 10.1016/j.urolonc.2022.03.004 35430138

