# Supplementary material for: Intermediate and high-risk non-muscle-invasive bladder cancer: an overview of epidemiology, burden, and unmet needs
Source: Front Oncol. 2023 Jun 2;13:1170124. doi: 10.3389/fonc.2023.1170124 (PMC10272547; doi:10.3389/fonc.2023.1170124)
Supplement: Supplementary file 1 [file DataSheet_1.pdf]

Supplementary Material

Intermediate and High-Risk Non-Muscle-Invasive Bladder Cancer: An Overview of Epidemiology, Burden, and Unmet Needs

Kristin Grabe-Heyne, Christof Henne, Paramananthan Mariappan, Götz Geiges, Johannes Pöhlmann, Richard F. Pollock*

*** Correspondence:** Richard F. Pollock, pollock@covalence-research.com

1 High-Quality Treatment Guidelines

Treatment guidelines considered to be “strongly recommended” by Maisch *et al.* (1) are summarised in Table S1 or, in the case of the National Institute for Health and Care Excellence, in Table S2. Additional guidelines are summarised in Table S2.

Table S1 High-Quality Treatment Guidelines

EAU	AUA/SUO	NCCN
2021 (2)	2016, amended 2020 (3)	2022 (version 2.2022) (4)
TURBT		
Recommended, followed by pathology investigation	Recommended	Recommended
Single, immediate, postoperative intravesical CT		
For low-risk tumors or small papillary recurrence detected ≥1 after TURBT	Consider in low- or intermediate-risk tumors	Recommended, within 24 hours of TURBT
Second TURBT		
Initial TURBT incomplete<br>No detrusor muscle in the specimen on initial resection, except for Ta LG/G1 and primary Cis<br>T1 tumor	Initial TURBT incomplete<br>T1 tumors<br>Consider for high-risk HG Ta tumors	Initial TURBT incomplete<br>No muscle in the original specimen in case of high-grade disease<br>Large (≥3 cm) or multifocal disease<br>T1 tumor
Treatment of primary or BCG-naïve tumors: low-risk tumors		
Single CT instillation considered the standard and complete treatment	Consider single CT instillation, no further treatment	No further treatment

EAU	AUA/SUO	NCCN
Treatment of primary or BCG-naïve tumors: intermediate-risk tumors		
1-year full-dose BCG (induction and 3-weekly instillations at 3, 6, and 12 months) or<br>Intravesical CT for ≤1 year (optimal schedule unknown)	Consider 6-week induction of intravesical CT or BCG and, if response, of maintenance therapy (BCG up to 1 year)	Intravesical therapy preferred (induction and maintenance with BCG or CT), no standard regimen for BCG
Treatment of primary or BCG-naïve tumors: high- and very high-risk tumors		
Full-dose BCG over 1 to 3 years (induction and 3-weekly instillations at 3, 6, 12, 18, 24, 30, and 36 months)<br>Weigh benefits of 2nd and 3rd maintenance year against costs, side effects, and BCG shortage<br>Discuss immediate RC	Give 6-week induction of BCG and, if complete response, 3-year maintenance with BCG	If very high-risk features: cystectomy preferred, BCG (induction and maintenance) as alternative<br>If no very high-risk features: BCG (induction and maintenance) preferred, cystectomy as alternative
Treatment following the failure of prior intravesical CT		
Patients can benefit from BCG instillations	In intermediate- and high-risk with persistent or recurrent disease or positive cytology following intravesical therapy, consider prostatic urethral biopsy and upper tract evaluation before additional intravesical therapy	As for the treatment of BCG failure

EAU	AUA/SUO	NCCN
Treatment following the failure of BCG		
RC<br>If not a candidate for RC, offer preservation strategies (e.g., intravesical CT, electromotive administration of chemotherapy, systemic immunotherapy), preferable as part of clinical trials	In intermediate- and high-risk with persistent or recurrent disease or positive cytology following intravesical therapy, consider prostatic urethral biopsy and upper tract evaluation before additional intravesical therapy<br>Offer second BCG course after single induction BCG course in intermediate- and high-risk with persistent or recurrent Ta or Cis<br>If HG T1 after single induction BCG course and fit for surgery, offer RC<br>If RC is not an option, consider a clinical trial or, if unavailable, intravesical CT or systemic immunotherapy	RC<br>Change of intravesical agent (valrubicin approved for BCG-refractory Cis)<br>Pembrolizumab in select patients

Abbreviations: BCG, Bacillus Calmette-Guérin; Cis, Carcinoma in situ; CT, Chemotherapy; EMDA, Electromotive Drug Administration; HG, High-Grade; LG, Low-Grade; NMIBC, Non-Muscle-Invasive Bladder Cancer; RC, Radical Cystectomy; US, United States.

Note: Updated from Shore et al. (5).

2 Treatment Guidelines from Canada and Selected European Countries

Table S2 Canadian and Selected European NMIBC treatment guidelines

**Canada**<br><br>**CUA**	**France**<br><br>**ccAFU**	**Germany**<br><br>**S3**	**Germany, Austria, Switzerland**<br><br>**Onkopedia**	**UK**<br><br>**NICE**
2021, Bhindi *et al.* (6)	2020–2022, Rouprêt *et al.* (7)	2020, Leitlinienprogramm Onkologie (8)	2019, de Wit *et al.* (9)	2015, NICE (10)
TURBT				
Recommended	Recommended	Recommended	Recommended	Recommended
Single, immediate, postoperative intravesical CT				
Should be offered to all low- and intermediate-risk patients (for intermediate risk even if further adjuvant CT is planned)<br>Benefit in high-risk disease unclear if intravesical BCG planned	For low-risk tumors and is an option in intermediate-risk disease	Can be performed unless contraindicated, highest benefit in primary, unifocal, and low-risk disease	Can be considered in low-risk patients	Offer to all bladder cancer cases at the time of first TURBT
Second TURBT if:				
Initial TURBT incomplete<br>T1 tumors<br>Consider in select HG Ta tumors	Initial TURBT not complete<br>In high-risk disease (T1 and/or G3 and/or Cis) and very-high risk	Initial TURBT incomplete<br>No detrusor muscle in the specimen (except in Ta LG)	Initial TURBT incomplete<br>No detrusor muscle in the specimen (except Ta)<br>T1 tumors	No detrusor muscle in the specimen<br>High-risk disease identified in first TURBT

Canada<br><br>CUA	France<br><br>ccAFU	Germany<br><br>S3	Germany, Austria, Switzerland<br><br>Onkopedia	UK<br><br>NICE
Not required if initial TURBT indicates RC	disease if not directly proceeding to RC	T1 tumors<br>HG tumors (except Cis)	High-risk, except Cis	
Treatment of primary or BCG-naïve tumors: low-risk tumors				
Single CT instillation	Single CT instillation	Single CT instillation	Single CT instillation can be considered	Single CT instillation

Canada<br><br>CUA	France<br><br>ccAFU	Germany<br><br>S3	Germany, Austria, Switzerland<br><br>Onkopedia	UK<br><br>NICE
Treatment of primary or BCG-naïve tumors: intermediate-risk tumors				
Consider adjuvant induction of intravesical CT (with subsequent maintenance up to 1 year) or induction BCG with maintenance therapy (1 year, weekly instillations for 3 weeks at 3, 6, 12 months)<br>If second-line BCG; consider reduced dosing during induction in case of BCG shortage<br>Stratification possible for recurrent LG Ta, into low-intermediate (treat as low-risk) and high-intermediate (treat as high-risk) based on tumor number and size, time to recurrence, and recurrence frequency	Intravesical CT (induction and 1-year maintenance) is usually offered as first-line due to better tolerability and higher effectiveness relative to BCG<br>BCG (6-week induction and 1-year maintenance)	BCG (6-week induction and 1-year maintenance with three weekly instillations at 3, 6, and 12 months)<br>Maintenance therapy with intravesical CT is an alternative to BCG	Intravesical CT (mitomycin-C) or BCG over 1 to 3 years	Offer ≥6 doses of intravesical mitomycin-C
Treatment of primary or BCG-naïve tumors: high- and very high-risk tumors				
BCG with induction (weekly for 6 weeks) and maintenance (weekly instillations for 3 weeks	BCG (6-week induction and 3-year maintenance)	Early RC (including as an alternative to BCG in solitary Cis)	Intravesical BCG over 1 to 3 years<br>Also, consider early RC	RC or BCG (induction and maintenance)

Canada<br>CUA	France<br>ccAFU	Germany<br>S3	Germany, Austria, Switzerland<br>Onkopedia	UK<br>NICE
after 3, 6, 12, 18, 24, 30, and 36 months)<br>Consider dosing reductions and/or shortening maintenance to 1 year in case of BCG shortage<br>Offer upfront RC to patients with large-volume, diffuse, endoscopically unresectable NMIBC or HG T1 with additional adverse tumor pathology	RC can be proposed as first-line treatment in very-high risk disease	If no early RC, use BCG (with maintenance of 1 to 3 years [three weekly instillations after 3, 6, 12, 18, 24, 30, 36 months]) if complete remission after the induction phase)		
Treatment following the failure of prior intravesical CT				
Offer induction and maintenance BCG	Induction and maintenance BCG	Not specified	Not specified	Not specified

Canada<br>CUA	France<br>ccAFU	Germany<br>S3	Germany, Austria, Switzerland<br>Onkopedia	UK<br>NICE
Treatment following the failure of BCG				
RC with pelvic lymph node dissection in surgically fit patients<br>Consider second-line bladder-preserving therapy before RC in BCG-unresponsive Cis or HG Ta (including pembrolizumab, intravesical oportuzumab monatox, nadofaragene firadenovec, and BCG plus N-803 for BCG-unresponsive Cis). Alternatives include sequential intravesical gemcitabine/docetaxel (induction and maintenance) or other combination or single agent intravesical therapy if RC is not an option.	Chemohyperthermia can be considered in case of BCG failure (or shortage) but has inferior oncologic outcomes	RC (in high-risk patients)	RC (in high-risk patients)<br>Offer multimodal primary bladder-sparing therapy if RC is not feasible or refused	Refer patient to specialist urology multidisciplinary team<br>Consider fulguration for recurrent disease without prior intermediate- or high-risk disease, a disease-free interval of ≥6 months, solitary papillary recurrence, tumor diameter ≤3 mm

Abbreviations: BCG, Bacillus Calmette-Guérin; ccAFU, Comité de Cancérologie de l'Association Française d'Urologie; Cis, Carcinoma in situ; CT, Chemotherapy; CUA, Canadian Urological Association; HG, High-Grade; LG, Low-Grade; NICE, National Institute for Health and Care Excellence; NMIBC, Non-Muscle-Invasive Bladder Cancer; RC, Radical Cystectomy; UK, United Kingdom.

3 Treatment Compliance Rate

Table S3 Treatment Compliance Rates

Study, study design	Single, immediate CT instillation	Second TURBT	Intravesical therapy, intermediate-risk NMIBC	Intravesical BCG, high-risk NMIBC	RC, highest risk NMIBC
Choo *et al.* (11), online survey of 701 members of three Asian urology societies	>90% of time: 48%<br>Selectively: 42%<br>Never: 10%	In pT1 or high-grade NMIBC:<br>>50% of the time: 49% >10% of the time: 79%	Not reported	Maintenance therapy:<br>≥90% of the time: 30%<br>≥50% of the time: 12%	Not reported
Hendricksen *et al.* (12), online survey of 498 urologists and urooncologists from nine European countries	IR: 60%<br>HR: 53%	HR: 80%	57% (CT or BCG for 1 year)	For 1 year: 38%<br>For 3 years: 41%	Not reported
Jeglinischi *et al.* (13), clinical record review of 159 French patients (30% low, 18% intermediate, 44% high, 8% very high risk)	Not reported	HR: 37% (of those indicated for second TURBT)	14% (all CT)	39% (with an average of 8.1 instillations per patient)	8% (of those indicated for RC)
Mariappan *et al.* (14), data covering 2,689 patients from collaborating centers in Scotland (4,246 consecutive patients in all of Scotland)	Scotland: 67, 68, and 67%; study cohort: 76, 69, and 74%; in quality performance indicator years 1 to 3	Not reported	Not reported	Not reported	Not reported

Study, study design	Single, immediate CT instillation	Second TURBT	Intravesical therapy, intermediate-risk NMIBC	Intravesical BCG, high-risk NMIBC	RC, highest risk NMIBC
Miyake *et al.* (15), retrospective, hospital claims-based study of 6,140 patients in Japan with ≥1 BCG prescription and ≥1 record at ≥12 weeks after the initial BCG dose	Not reported	Not reported	Completed BCG induction: 75%<br>Did not complete BCG induction: 75%		Not reported
Reis *et al.* (16), survey of 476 attendants of the Brazilian Congress of Urology	HR: 4.6%	67.6%	Not reported	HR: 78.8% (75.6% [of entire sample] reporting use of maintenance therapy)	Not reported (9.7% in high-risk disease)
Sountoulides *et al.* (17), clinical audit of 101 incident bladder cancer cases in the UK	49.3%	54.3%	Not reported	Not reported	Not reported
Tobert *et al.* (18), database review of 847 US patients with high-grade NMIBC	28.2%	Not reported	≥6 BCG instillations: 56%		Not reported
Wang *et al.* (19), online survey of 814 Chinese urologists	Not reported	After incomplete initial TURBT: 67.4%<br>No detrusor muscle in specimen: 74.0%	93.0% (induction and maintenance)	Induction and maintenance:<br>IR: 67.6%<br>HR: 80.7%	High-grade T1 with histological variation: 50.0%<br>High-grade T1 with lymphatic infiltration,

Study, study design	Single, immediate CT instillation	Second TURBT	Intravesical therapy, intermediate-risk NMIBC	Intravesical BCG, high-risk NMIBC	RC, highest risk NMIBC
		In T1 tumors: 44.4%<br>In G3/high-grade tumors except for CIS: 54.9%			multiple/large lesions, or CIS: 79.7%<br>High-grade T1 after second TURBT: 68.5%<br>High-grade NMIBC with recurrence within 3 months: 68.1%<br>High-risk NMIBC with BCG failure: 66.1%

Abbreviations: BCG, Bacillus Calmette-Guérin; CIS, Carcinoma In Situ; CT, Chemotherapy; HR, High Risk; IR, Intermediate Risk; NMIBC, Non-Muscle-Invasive Bladder Cancer; RC, Radical Cystectomy; SUO, Society of Urologic Oncology.

4 Reasons for Non-Compliance with Treatment Guidelines

Table S4 Reasons for Non-Compliance with Treatment Guidelines

Study, study design	Reasons for/factors influencing non-compliance
Lack of knowledge and/or training	
Balakrishnan *et al.* (20),<br>analysis of cT1 bladder cancer cases from the US National Cancer Database	Treatment in a community cancer center is associated with a higher risk of non-guideline-compliant therapy than in an academic center.

Study, study design	Reasons for/factors influencing non-compliance
Choo *et al.* (11)	• Second TURBT if no detrusor muscle in the first TURBT specimen is more likely in academic teaching than in private or non-teaching hospitals and more likely for surgeons with shorter than with longer practice (no statistically significant difference for either group regarding the second TURBT for T1 grade or high-grade disease)<br>• Single immediate chemotherapy instillation more likely in academic teaching than in private or non-teaching hospitals for T1 and high-grade disease (no statistically significant difference for other indications)<br>• Reported country-specific differences (between Japan, Korea, and Taiwan) that could not readily be explained
Jeglinschi *et al.* (13)	Guideline deviation based on a decision by treating urologist without reasons specified (of all guideline deviations in the risk group):<br>• Intermediate-risk patients not receiving second TURBT: 100% (one patient)<br>• High-risk patients not receiving second TURBT: 65.0%<br>• Intermediate-risk patients not starting intravesical therapy: 79.0%<br>• High-risk patients not starting intravesical therapy: 53.5%<br>• Very high-risk patients not receiving radical cystectomy: 18.2%
Matulay *et al.* (21), online survey of 121 urologists in the US	• Higher rates of guideline compliance in urologic oncologists versus non-urologic oncologists and in fellowship-trained versus non-fellowship-trained urologists<br>• Higher rates of guideline compliance among physicians based in academic relative to hospital employment and among physicians with shorter relative to longer experience

**Study, study design**	**Reasons for/factors influencing non-compliance**
Tobert *et al.* (18)	Guideline compliance is more likely if treated in an academic cancer center relative to non-academic, non-cancer centers
Lack of access and economic barriers	
Balakrishnan *et al.* (20)	Lack of insurance is associated with a higher risk of non-guideline-compliant therapy than being privately or Medicare-insured<br>Living in areas with lower adult educational attainment is associated with a higher risk of non-guidelines-compliant therapy
Choo *et al.* (11)	BCG shortage is the main reason for not giving maintenance BCG, particularly in Taiwan (less so in Japan, which has a more stable supply)
Jeglinschi *et al.* (13)	Guideline deviation due to lack of resources (of all guideline deviations):<br>• High-risk patients not receiving second TURBT: 2.3%
Wang *et al.* (19)	• 75% of guideline deviations for BCG are due to inaccessible BCG<br>• 50.4% of indicated second TURBTs, 40.7% of indicated BCG therapies, and 29.2% of indicated radical cystectomies were rejected by patients for economic reasons
Fear of complications or patient comorbidities	
Jeglinschi *et al.* (13)	Guideline deviation due to poor patient health status (of all guideline deviations in risk group):<br>• High-risk patients not receiving second TURBT: 20.0%<br>• High-risk patients not starting intravesical therapy: 23.3%<br>• Very high-risk patients not receiving second TURBT: 60.0%<br>• Very high-risk patients not receiving radical cystectomy: 63.6%
Wang *et al.* (19)	• 37.6% of indicated second TURBTs were not performed because the urologist was concerned about the risk of side effects; in 70.6%, the patient expressed such concerns<br>• 26.0% of indicated BCG therapies were not performed because the urologist was concerned about the risk of side effects; in 62.2%, patients expressed such concerns

Study, study design	Reasons for/factors influencing non-compliance
	• 38.1% of indicated radical cystectomies were rejected by patients concerned about side effects, while 57.7% were rejected by patients out of concerns for decreases in quality of life (47.4% rejected due to “personal reasons”)

5 Treatment Discontinuation in Patients with NMIBC

Table S5 Treatment Completion/Discontinuation in Patients with NMIBC

Study, study design	Findings on treatment completion
Abushamma *et al.* (22), histopathological review of 88 patients with NMIBC in Palestine	• Compliance with cystoscopic surveillance protocol: 23%<br>• For the non-compliant versus compliant group, statistically significantly higher rates, over 3 years, of:<br>o Recurrence: 55 versus 93%<br>o Progression: 5 versus 54%<br>o Metastasis: 5 versus 38%<br>o Mortality: 0 versus 24%
Alhogbani *et al.* (23), chart review of 303 French patients with an initial course of BCG	• Discontinuation patterns<br>o <6 instillations: 18%<br>▪ Discontinuation due to BCG shortage (40%) and local (31%) or systemic toxicity (29%)<br>o Completed induction but discontinued maintenance: 53%<br>▪ Discontinuation due to BCG shortage (60%) and grade II (31%) or III (8%) toxicity<br>o Completed induction and maintenance: 29%

**Study, study design**	**Findings on treatment completion**
	• Statistically significantly higher risk of recurrence, progression, and bladder cancer-specific mortality in non-completers compared to completers
Datovo *et al.* (24), chart review of 198 Brazilian patients with high-risk NMIBC	• 3-year adherence to cystoscopy protocol: 18%<br>• Statistically significantly higher progression risk in non-adherent patients (33.3% versus 17.9%, p=0.014), but no statistically significant differences for recurrence or mortality
Gontero *et al.* (25), clinical records from 2,451 patients with T1G3 NMIBC receiving BCG	• Planned BCG treatment completed: 76.5%<br>• Discontinuation due to local (3.4%) or systemic toxicity (1.3%)
Jeglinschi *et al.* (13)	Poor patient compliance is the reason for:<br>• High-risk patients not starting BCG: 21%<br>• High-risk patients not finishing BCG maintenance: 35%<br>• High-risk patients not undergoing second TURBT: 15%<br>• Very high-risk patients not undergoing radical cystectomy: 18%
Nummi *et al.* (26), chart review of 418 Finnish patients with NMIBC receiving BCG	• Interrupted BCG treatment: 42%<br>○ Due to adverse effects other than BCG infection (17%), BCG failure (11%), other including BCG shortage (9%), suspected BCG infection (6%)<br>• Only 81% of patients with interrupted treatment went on to standard surveillance (compared to 93% in those without treatment interruption); 63% of those with interrupted treatment underwent radical cystectomy
Oddens *et al.* (27), randomized controlled trial in 1,355 patients with intermediate- and high-risk NMIBC	• 1-year treatment in those randomized to 1-year treatment with one-third dose: 58%<br>• 1-year treatment in those randomized to 1-year treatment with full dose: 62%<br>• 3-year treatment in those randomized to 3-year treatment with one-third dose: 34%<br>• 3-year treatment in those randomized to 3-year treatment with full dose: 35%

Study, study design	Findings on treatment completion
Serretta *et al.* (28), cohort study of 411 Italian patients with T1HG NMIBC indicated for 1-year BCG treatment	• Patients stopping induction: 8% (refusal: 29%, local grade II or III toxicity: 19%, systemic toxicity: 16%)<br>• Patients starting maintenance: 75%, of whom 23% stopped maintenance early (mild toxicity that affects social life: 59%, concomitant disease: 16%, local toxicity: 10%)
Tapiero *et al.* (29), chart review for 729 Israeli patients with NMIBC receiving intravesical therapy	• Completing induction therapy:<br>o MMC: 87%<br>o BCG: 86%<br>• Completing maintenance therapy:<br>o MMC: 47%<br>o BCG: 10% with SWOG protocol, 55% with monthly instillation

Abbreviations: BCG, Bacillus Calmette-Guérin; MMC, Mitomycin-C; NMIBC, Non-Muscle-Invasive Bladder Cancer; TURBT, Transurethral Resection of Bladder Cancer.

6 Validation Results for Risk Scoring Models

Table S6 Validation Results for Risk Scoring Models/Tables

Model/risk table	Validation findings
AUA/SUO risk stratification (3)	Successful risk stratification but the limited predictive ability for progression and recurrence (validated multi-center phase 2 study from the US) (30)
CUETO model (31)	• CUETO model underestimates 5-year recurrence risk in low-risk patients and overestimates 5-year progression risk in high-risk patients (validated in EORTC data) (32)<br>• CUETO risk table is less successful than the EORTC risk table at discriminating risk groups (validated in a single-institution cohort from Turkey) (33)<br>• CUETO model successfully stratifies recurrence risk groups (validated in a single-institution cohort from the US) (34)

Model/risk table	Validation findings
	• CUETO model underestimates 1- and 5-year recurrence and progression risks (validated in Swedish cohort) (35)<br>• CUETO model underestimates 1-year recurrence and progression risks and overestimates 5-year progression risks (validated in two cohorts from Spanish and Polish institutions) (36)<br>• CUETO model has poor discrimination for recurrence and progression and overestimates risks for either outcome in high-risk patients, including in BCG-treated patients (validated in an international multi-center study) (37)<br>• CUETO model can stratify risks but has poor discrimination for predicting clinical events (validated in a multi-institutional cohort) (38)
EORTC (39)	• EORTC model underestimates 1- and 5-year recurrence and progression risks (validated in Swedish cohort) (35)<br>• EORTC model has poor discrimination for recurrence and progression and overestimates risks for either outcome in high-risk patients, including in BCG-treated patients (validated in an international multi-center study) (37)<br>• EORTC model stratifies recurrence and progression risk but has reduced discriminative ability for progression in patients treated with BCG (validated in CUETO data) (40)<br>• EORTC model performs better than CUETO and EAU pre-2021 models for predicting recurrence, progression, and mortality, but overall performance is modest (validated in a single-institution cohort from Poland) (41)

Model/risk table	Validation findings
EAU 2021 model (42)	• EAU 2021 model underestimates 1- and 5-year recurrence and progression risks (validated in Swedish cohort) (35)<br>• EUA 2021 model can stratify risks but has poor discrimination for predicting clinical events (validated in a multi-institutional cohort) (38)<br>• EAU 2021 prognostic risk factor groups stratify progression risk appropriately but overestimate progression risk in patients receiving BCG (validated single-institution cohort from the US) (43)<br>• EAU 2021 model introduces a very high-risk group that allows identifying patients more likely to progress but overall model accuracy is limited in patients with repeat transurethral resection of the bladder and BCG (assessed in a mult-institutional cohort from Italy) (44)

Abbreviations: AUA, American Urological Association; BCG, Bacillus Calmette-Guérin; CUETO, Spanish Urology Association for Oncological Treatment; EORTC, European Organisation for Research and Treatment of Cancer; SUO, Society for Urologic Oncology.

7 References

1. Maisch P, Retz M, Gschwend JE, Koll F, Schmid SC. Clinical practice guidelines for bladder cancer: a systematic review and meta-analysis using the AGREE II instrument. *Urol Int* (2021) 105:31–40. doi: 10.1159/000509431

2. Babjuk M, Burger M, Capoun O, Cohen D, Compérat EM, Dominguez Escrig JL, et al. European Association of Urology guidelines on non-muscle-invasive bladder cancer (Ta, T1, and carcinoma in situ). *Eur Urol* (2022) 81:75–94. doi: 10.1016/j.eururo.2021.08.010

3. Chang SS, Boorjian SA, Chou R, Clark PE, Daneshmand S, Konety BR, et al. Diagnosis and treatment of non-muscle invasive bladder cancer: AUA/SUO guideline, 2016; amended 2020. (2020) https://www.auanet.org/documents/education/clinical-guidance/Non-Muscle-Invasive-Bladder-Cancer.pdf [Accessed February 3, 2023]

4. Flaig TW, Spiess PE, Abern M, Agarwal N, Bangs R, Boorjian SA, et al. NCCN Clinical Practice Guidelines in Oncology (NCCN Guidelines®): bladder cancer. Plymouth Meeting, PA: National Comprehensive Cancer Network (2022).

5. Shore ND, Palou Redorta J, Robert G, Hutson TE, Cesari R, Hariharan S, et al. Non-muscle-invasive bladder cancer: an overview of potential new treatment options. *Urol Oncol* (2021) 39:642–663. doi: 10.1016/j.urolonc.2021.05.015

6. Bhindi B, Kool R, Kulkarni GS, Siemens DR, Aprikian AG, Breau RH, et al. Canadian Urological Association guideline on the management of non-muscle-invasive bladder cancer. *Can Urol Assoc J* (2021) 15:E424–E460. doi: 10.5489/cuaj.7367

7. Rouprêt M, Pignot G, Masson-Lecomte A, Compérat E, Audenet F, Roumiguié M, et al. French ccAFU guidelines - update 2020-2022: bladder cancer. *Prog En Urol* (2020) 30:S78–S135. doi: 10.1016/S1166-7087(20)30751-X

8. Leitlinienprogramm Onkologie (Deutsche Krebsgesellschaft, Deutsche Krebshilfe, AWMF). S3-Leitlinie Früherkennung, Diagnose, Therapie und Nachsorge des Harnblasenkarzinoms, Langversion 2.0. Berlin: Leitlinienprogramm Onkologie (2020). https://www.leitlinienprogramm-onkologie.de/leitlinien/harnblasenkarzinom/ [Accessed August 5, 2022]

9. de Wit M, Bauernhofer T, Bokemeyer C, Grimm M-O, Hoffmann W, Lorch A, et al. Blasenkarzinom (Urothelkarzinom): Leitlinie. Deutsche Gesellschaft für Hämatologie und Medizinische Onkologie and Österreiche Gesellschaft für Hämatologie & Medizinische Onkologie and Schweizerische Gesellschaft für Medizinische Onkologie and Société Suisse d'Hématologie (2019). https://www.onkopedia.com/de/onkopedia/guidelines/blasenkarzinom-urothelkarzinom/@@guideline/html/index.html [Accessed August 6, 2022]

10. NICE. Bladder cancer: diagnosis and management. National Institute for Health and Care Excellence (2015). https://www.nice.org.uk/guidance/ng2 [Accessed March 8, 2023]

11. Choo SH, Nishiyama H, Kitamura H, Chen C-H, Pu Y-S, Lee H-L, et al. Practice pattern of non-muscle invasive bladder cancer in Japan, Korea and Taiwan: a web-based survey. *Int J Urol* (2019) 26:1121–1127. doi: 10.1111/iju.14105

12. Hendricksen K, Aziz A, Bes P, Chun FK-H, Dobruch J, Kluth LA, et al. Discrepancy between European Association of Urology guidelines and daily practice in the management of non-muscle-invasive bladder cancer: results of a European survey. *Eur Urol Focus* (2019) 5:681–688. doi: 10.1016/j.euf.2017.09.002

13. Jeglinschi S, Schirmann A, Durand M, Sanchez S, Larré S, Léon P. Factors affecting guideline adherence in the initial treatment of non-muscle invasive bladder cancer: Retrospective study in a French peripheral hospital. *Prog En Urol* (2020) 30:26–34. doi: 10.1016/j.purol.2019.11.003

14. Mariappan P, Johnston A, Padovani L, Clark E, Trail M, Hamid S, et al. Enhanced quality and effectiveness of transurethral resection of bladder tumour in non-muscle-invasive bladder cancer: a multicentre real-world experience from Scotland's Quality Performance Indicators Programme. *Eur Urol* (2020) 78:520–530. doi: 10.1016/j.eururo.2020.06.051

15. Miyake M, Kikuchi E, Shinozaki K, Piao Y, Hayashi N, Koto R, et al. Real-world treatment patterns and clinical outcomes of Japanese patients with non-muscle invasive bladder cancer receiving intravesical bacillus Calmette-Guérin treatment. *Int J Urol* (2022) 29:1120–1129. doi: 10.1111/iju.14933

16. Reis LO, Moro JC, Ribeiro LFB, Voris BRI, Sadi MV. Are we following the guidelines on non-muscle invasive bladder cancer? *Int Braz J Urol* (2016) 42:22–28. doi: 10.1590/S1677-5538.IBJU.2015.0122

17. Sountoulides P, Mutomba WF, Bouras E, Lim J, Bourdoumis A, Jain A. How well do we manage non-muscle invasive bladder tumors? A UK audit of real-life practices. *Urologia* (2020) 87:142–148. doi: 10.1177/0391560319899303

18. Tobert CM, Nepple KG, McDowell BD, Charlton ME, Mott SL, Gruca TS, et al. Compliance with American Urological Association guidelines for nonmuscle invasive bladder cancer remains poor: assessing factors associated with noncompliance and survival in a rural state. *Urology* (2019) 132:150–155. doi: 10.1016/j.urology.2019.06.021

19. Wang D-Q, Huang Q, Huang X, Jin Y-H, Wang Y-Y, Shi Y-X, et al. Knowledge of and compliance with guidelines in the management of non-muscle-invasive bladder cancer: a survey of Chinese urologists. *Front Oncol* (2021) 11:735704. doi: 10.3389/fonc.2021.735704

20. Balakrishnan AS, Washington SL, Meng MV, Porten SP. Determinants of guideline-based treatment in patients with cT1 bladder cancer. *Clin Genitourin Cancer* (2019) 17:e461–e471. doi: 10.1016/j.clgc.2019.01.007

21. Matulay JT, Tabayoyong W, Duplisea JJ, Chang C, Daneshmand S, Gore JL, et al. Variability in adherence to guidelines based management of nonmuscle invasive bladder cancer among Society of Urologic Oncology (SUO) members. *Urol Oncol* (2020) 38:796.e1-796.e6. doi: 10.1016/j.urolonc.2020.04.026

22. Abushamma F, Khayyat Z, Soroghle A, Zyoud SH, Jaradat A, Akkawi M, et al. The impact of non-compliance to a standardized risk-adjusted protocol on recurrence, progression, and mortality in non-muscle invasive bladder cancer. *Cancer Manag Res* (2021) 13:2937–2945. doi: 10.2147/CMAR.S299148

23. Alhogbani MM, Picard JA, Fassi-Fehri MH, Badet JL, Colombel CM. Prognostic impact of Bacillus Calmette-Guérin interruption at the time of induction and consolidation. *Urol Ann* (2017) 9:315–320. doi: 10.4103/UA.UA_115_17

24. Datovo JCF, Neto WA, Mendonça GB, Andrade DL, Reis LO. Prognostic impact of non-adherence to follow-up cystoscopy in non-muscle-invasive bladder cancer (NMIBC). *World J Urol* (2019) 37:2067–2071. doi: 10.1007/s00345-019-02697-8

25. Gontero P, Sylvester R, Pisano F, Joniau S, Vander Eeckt K, Serretta V, et al. Prognostic factors and risk groups in T1G3 non-muscle-invasive bladder cancer patients initially treated with Bacillus Calmette-Guérin: results of a retrospective multicenter study of 2451 patients. *Eur Urol* (2015) 67:74–82. doi: 10.1016/j.eururo.2014.06.040

26. Nummi A, Järvinen R, Sairanen J, Huotari K. A retrospective study on tolerability and complications of bacillus Calmette-Guérin (BCG) instillations for non-muscle-invasive bladder cancer. *Scand J Urol* (2019) 53:116–122. doi: 10.1080/21681805.2019.1609080

27. Oddens J, Brausi M, Sylvester R, Bono A, van de Beek C, van Andel G, et al. Final results of an EORTC-GU cancers group randomized study of maintenance bacillus Calmette-Guérin in intermediate- and high-risk Ta, T1 papillary carcinoma of the urinary bladder: one-third dose versus full dose and 1 year versus 3 years of maintenance. *Eur Urol* (2013) 63:462–472. doi: 10.1016/j.eururo.2012.10.039

28. Serretta V, Scalici Gesolfo C, Alonge V, Cicero G, Moschini M, Colombo R. Does the compliance to intravesical BCG differ between common clinical practice and international multicentric trials? *Urol Int* (2016) 96:20–24. doi: 10.1159/000430501

29. Tapiero S, Helfand A, Kedar D, Yossepowitch O, Nadu A, Baniel J, et al. Patient compliance with maintenance intravesical therapy for nonmuscle invasive bladder cancer. *Urology* (2018) 118:107–113. doi: 10.1016/j.urology.2018.04.039

30. Ritch CR, Velasquez MC, Kwon D, Becerra MF, Soodana-Prakash N, Atluri VS, et al. Use and validation of the AUA/SUO risk grouping for nonmuscle invasive bladder cancer in a contemporary cohort. *J Urol* (2020) 203:505–511. doi: 10.1097/JU.0000000000000593

31. Fernandez-Gomez J, Madero R, Solsona E, Unda M, Martinez-Piñeiro L, Gonzalez M, et al. Predicting nonmuscle invasive bladder cancer recurrence and progression in patients treated with bacillus Calmette-Guerin: the CUETO scoring model. *J Urol* (2009) 182:2195–2203. doi: 10.1016/j.juro.2009.07.016

32. Cambier S, Sylvester RJ, Collette L, Gontero P, Brausi MA, van Andel G, et al. EORTC nomograms and risk groups for predicting recurrence, progression, and disease-specific and overall survival in non–muscle-invasive stage Ta–T1 urothelial bladder cancer patients treated

with 1–3 years of maintenance Bacillus Calmette-Guérin. *Eur Urol* (2016) 69:60–69. doi: 10.1016/j.eururo.2015.06.045

33. Dalkilic A, Bayar G, Kilinc MF. A comparison of EORTC and CUETO risk tables in terms of the prediction of recurrence and progression in all non-muscle-invasive bladder cancer patients. *Urol J* (2019) 16:37–43. doi: 10.22037/uj.v0i0.4091

34. Rosevear HM, Lightfoot AJ, Nepple KG, O'Donnell MA. Usefulness of the Spanish Urological Club for Oncological Treatment scoring model to predict nonmuscle invasive bladder cancer recurrence in patients treated with intravesical bacillus Calmette-Guérin plus interferon-α. *J Urol* (2011) 185:67–71. doi: 10.1016/j.juro.2010.08.083

35. Dovey Z, Pfail J, Martini A, Steineck G, Dey L, Renström L, et al. Bladder cancer (NMIBC) in a population-based cohort from Stockholm County with long-term follow-up; A comparative analysis of prediction models for recurrence and progression, including external validation of the updated 2021 E.A.U. model. *Urol Oncol* (2022) 40:106.e1-106.e10. doi: 10.1016/j.urolonc.2021.10.008

36. Krajewski W, Rodríguez-Faba O, Breda A, Pisano F, Poletajew S, Tukiendorf A, et al. Validation of the CUETO scoring model for predicting recurrence and progression in T1G3 urothelial carcinoma of the bladder. *Actas Urol Esp* (2019) 43:445–451. doi: 10.1016/j.acuro.2019.02.006

37. Xylinas E, Kent M, Kluth L, Pycha A, Comploj E, Svatek RS, et al. Accuracy of the EORTC risk tables and of the CUETO scoring model to predict outcomes in non-muscle-invasive urothelial carcinoma of the bladder. *Br J Cancer* (2013) 109:1460–1466. doi: 10.1038/bjc.2013.372

38. Krajewski W, Aumatell J, Subiela JD, Nowak Ł, Tukiendorf A, Moschini M, et al. Accuracy of the CUETO, EORTC 2016 and EAU 2021 scoring models and risk stratification tables to predict outcomes in high-grade non-muscle-invasive urothelial bladder cancer. *Urol Oncol* (2022) 40:491.e11-491.e19. doi: 10.1016/j.urolonc.2022.06.008

39. Sylvester RJ, van der Meijden APM, Oosterlinck W, Witjes JA, Bouffioux C, Denis L, et al. Predicting recurrence and progression in individual patients with stage Ta T1 bladder cancer using EORTC risk tables: a combined analysis of 2596 patients from seven EORTC trials. *Eur Urol* (2006) 49:466–465; discussion 475-477. doi: 10.1016/j.eururo.2005.12.031

40. Fernandez-Gomez J, Madero R, Solsona E, Unda M, Martinez-Piñeiro L, Ojea A, et al. The EORTC tables overestimate the risk of recurrence and progression in patients with non-muscle-invasive bladder cancer treated with bacillus Calmette-Guérin: external validation of the EORTC risk tables. *Eur Urol* (2011) 60:423–430. doi: 10.1016/j.eururo.2011.05.033

41. Jobczyk M, Stawiski K, Fendler W, Różański W. Validation of EORTC, CUETO, and EAU risk stratification in prediction of recurrence, progression, and death of patients with initially non–muscle-invasive bladder cancer (NMIBC): A cohort analysis. *Cancer Med* (2020) 9:4014–4025. doi: 10.1002/cam4.3007

42. Sylvester RJ, Rodríguez O, Hernández V, Turturica D, Bauerová L, Bruins HM, et al. European Association of Urology (EAU) prognostic factor risk groups for non–muscle-invasive bladder cancer (NMIBC) incorporating the WHO 2004/2016 and WHO 1973 classification systems for grade: an update from the EAU NMIBC Guidelines Panel. *Eur Urol* (2021) 79:480–488. doi: 10.1016/j.eururo.2020.12.033

43. Lobo N, Hensley PJ, Bree KK, Nogueras-Gonzalez GM, Navai N, Dinney CP, et al. Updated European Association of Urology (EAU) prognostic factor risk groups overestimate the risk of progression in patients with non-muscle-invasive bladder cancer treated with Bacillus Calmette-Guérin. *Eur Urol Oncol* (2022) 5:84–91. doi: 10.1016/j.euo.2021.11.006

44. Contieri R, Hurle R, Paciotti M, Casale P, Saita A, Porpiglia F, et al. Accuracy of the European Association of Urology (EAU) NMIBC 2021 scoring model in predicting progression in a large cohort of HG T1 NMIBC patients treated with BCG. *Minerva Urol Nephrol* (2023) 75:180–187. doi: 10.23736/S2724-6051.22.04953-9
